# Licorice-Yuanhua Herbal Pair Induces Ileum Injuries Through Weakening Epithelial and Mucous Barrier Functions: Saponins, Flavonoids, and Di-Terpenes All Involved

**DOI:** 10.3389/fphar.2020.00869

**Published:** 2020-07-16

**Authors:** Jingao Yu, Dongbo Zhang, Yanni Liang, Zhen Zhang, Jianming Guo, Yanyan Chen, Yafeng Yan, Hongbo Liu, Liyan Lei, Zheng Wang, Zhishu Tang, Yuping Tang, Jin-ao Duan

**Affiliations:** ^1^Shaanxi Collaborative Innovation Center of Chinese Medicine Resources Industrialization, State Key Laboratory of Research & Development of Characteristic Qin Medicine Resources (Cultivation), Shaanxi Innovative Drug Research Center, The Youth Innovation Team of Shaanxi Universities, Shaanxi University of Chinese Medicine, Xianyang, China; ^2^Jiangsu Collaborative Innovation Center of Chinese Medicinal Resources Industrialization, National and Local Collaborative Engineering Center of Chinese Medicinal Resources Industrialization and Formulae Innovative Medicine, Jiangsu Key Laboratory for High Technology Research of TCM Formulae, Nanjing University of Chinese Medicine, Nanjing, China

**Keywords:** incompatible herbal pair, toxicity, gut barrier, licorice, *Daphne genkwa*

## Abstract

In traditional Chinese Medicine (TCM), the licorice-yuanhua herbal pair is one of the most representative incompatible herbal pairs recorded in the “eighteen incompatible herbal pairs” theory. Previous studies of our research group have demonstrated several gut-related side-effects induced by the licorice-yuanhua herbal pair. In this study, we investigated whether and why this incompatible herbal pair could induce gut tissue damage. After licorice-yuanhua treatment, the duodenum, ileum, and colon and serum biomarkers of mice were examined by pathological staining, Western blot, and ELISA assays. The IEC-6 cells and LS174T cells were treated with licorice saponins, yuanhua flavonoids, and di-terpenes; iTRAQ-labeled proteomic technology was then used to explore their synergistic effects on mucosa cells, followed by verification of ZO-1 and MUC-2 protein expressions. The results showed that the licorice-yuanhua herbal pair induced ileum tissue injuries, including epithelial integrity loss, inflammation, and edema. These injuries were verified to be related to epithelial and mucous barrier weakening, such as downregulated ileum ZO-1 and MUC-2 protein expressions. Proteomic analysis also suggested that glycyrrhizic acid and genkwanin synergistically influence tight junction pathways in LS174T cells. Furthermore, licorice saponins, yuanhua flavonoids, and di-terpenes dose/structure-dependently downregulate ZO-1 and MUC-2 protein expressions in mucosa cells. Our study provides different insights into the incompatibility mechanisms and material basis of the licorice-yuanhua herbal pair, especially that besides toxic di-terpenes, licorice saponins and yuanhua flavonoids, which are commonly known to be non-toxic compounds, can also take part in the gut damage induced by the licorice-yuanhua herbal pair.

## Introduction

In traditional Chinese medicine (TCM), the vast majority of herbal slices are used jointly in TCM prescriptions, leading to a wide range of herbal–herbal interactions *in vitro* and *in vivo* (Enioutina et al., 2017). The herbal–herbal interactions generate different types of herbal compatibility relations. These relations are called “Qi-Qing” in TCM classics, which means seven types of compatibility relations; one of the seven relations is “Xiang-Fan,” which means incompatible herbal pairs that could induce toxicity to the body under special conditions (Liu, 2010; Fan et al., 2015). In TCM, there are eighteen incompatible herbal pairs according to the “Eighteen Incompatibilities” theory taken from Ru Men Shi Qin, a Chinese ancient medicinal book of the Jin dynasty. The licorice-yuanhua herbal pair is one of the eighteen incompatible herbal pairs. Although there is a limited number of incompatible herbal pairs, the incompatibility mechanisms of these herbal pairs are very complicated, involving almost every stage of drug disposition processes *in vivo* (Tang et al., 2015). In the Chinese Pharmacopeia, these eighteen incompatible herbal pairs are prohibited in prescriptions, though several classical prescriptions used in clinic contain incompatible herbal pairs, such as Gansui-Banxia Decoction, Haizao-Yuhu Decoction, Gan-Zao Decoction, etc. (Shen et al., 2013; Ma et al., 2016; Yu et al., 2018). This confused situation is largely due to the ambiguity of the conditions of occurrence and interaction mechanisms of these incompatibilities.

Licorice, the dried roots of *Glycyrrhiza uralensis* Fisch., *Glycyrrhiza inflate* Bat., or *Glycyrrhiza glabra* L., is one of the most frequently used herbs in TCM clinics (Cai et al., 2011). TCM doctors believe that licorice has many benefits, including tonifying digestive systems and moderating the properties of different herbs, as well as detoxifying poisonous herbs. Licorice is commonly known as being very safe to the body, and it is widely used in food industries as a sweetener in cookies and other daily foods (Karaaslan and Dalgıç, 2014). However, when licorice is used jointly with yuanhua, the incompatible licorice-yuanhua herbal pair is believed to induce exacerbated toxic effects (Guo et al., 2014). Yuanhua is the dried flower bud of *Daphne genkwa* Sieb. et Zucc. It has some potential side effects on the body due to the toxic di-terpene compounds within it, but it is considered non-toxic at clinical doses (Jin et al., 2019). The main active compounds in licorice and yuanhua have been much clarified: the main active compounds in the aqueous extract of licorice are triterpenoid saponins such as glycyrrhizic acid (GA), which can be metabolized into glycyrrhetinic acid monoglucuronide (GAMG) and glycyrrhetinic acid (GRA) (Tao et al., 2013); for yuanhua, large amounts of flavonoids exist in its aqueous and alcohol extracts, including genkwanin (GKW), apigenin (APG), hydroxy genkwanin (HGKW), luteolin (LUT), luteolin glucoside (LUTG), and tiliroside (TLS). Additionally, di-terpenes such as yuanhuacine and yuanhuapine are considered as both toxic and active compounds (Chen et al., 2012; Chen et al., 2013a).

Our previous studies have proved that the licorice-yuanhua herbal pair can lead to gut irritation, liver or kidney toxicity, metabolic disorders, gut microflora dysbiosis, and colonic hydrogen sulfide overproduction when under clinical doses (Yu et al., 2017; Chen et al., 2019; Yu et al., 2019). Although the incompatibility of the licorice-yuanhua herbal pair has been verified by our research group, the mechanisms and damage-related compounds have not yet been fully uncovered.

**Figure 1:**
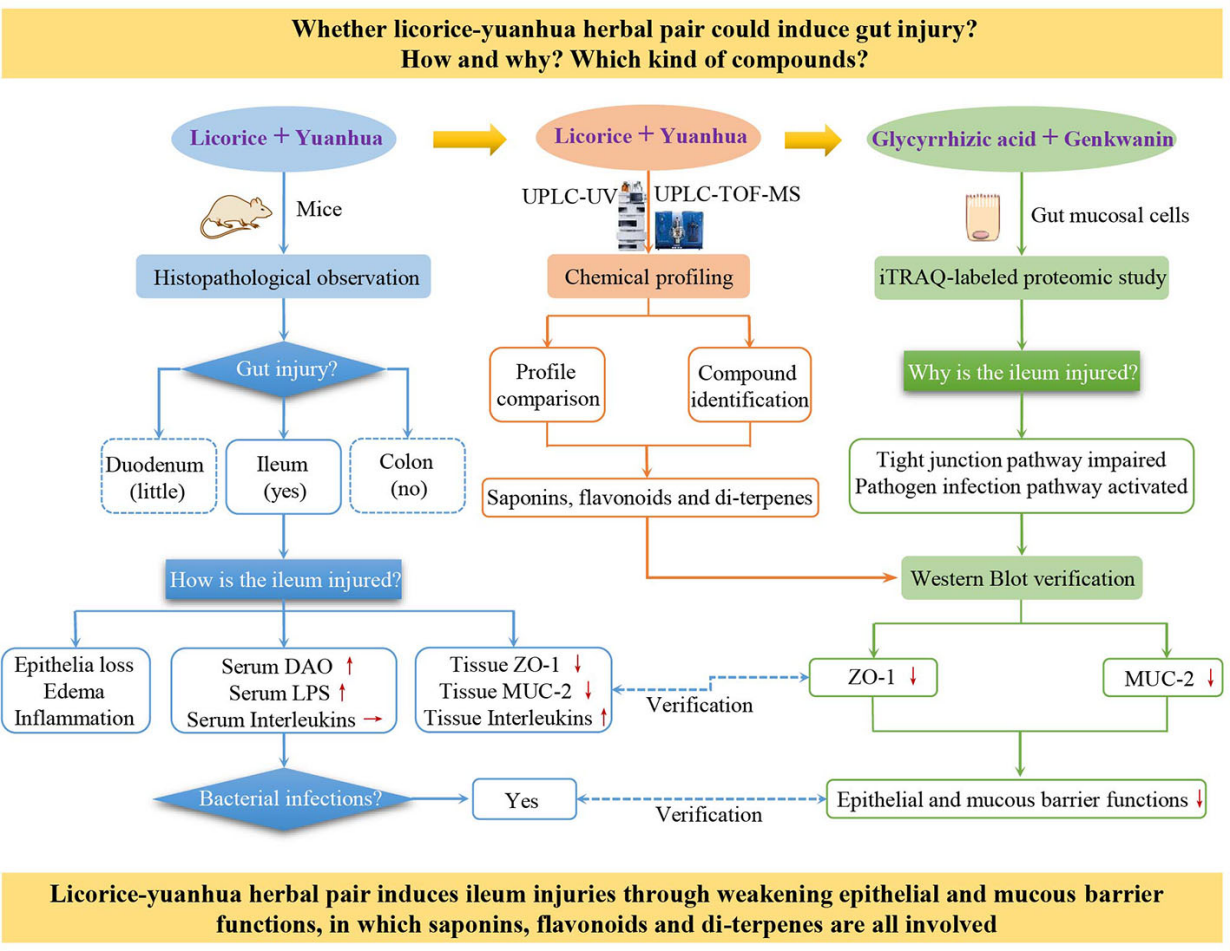
Research frame of this study. The “+” symbol means combination, red up and down-arrows mean increased and decreased, respectively, and a horizontal arrow means not changed.

In this study, considering the gut irritation, microflora dysfunction, and colonic hydrogen sulfide metabolic disorder induced by the licorice-yuanhua herbal pair, we further suppose that it is probable that this herbal pair induces gut tissue damage or functional impairment. Hence, mice were treated with the licorice-yuanhua herbal pair to verify our hypothesis. Then, mouse duodenum, ileum, and colon and serum biomarkers were examined by pathological staining, Western blot, and ELISA assays. iTRAQ-labeled proteomics experiments were then employed to explore the synergistic effects of licorice saponins and yuanhua flavonoids on mucosa cells, and lastly, licorice saponins, yuanhua flavonoids, and di-terpenes were examined with IEC-6 cells and LS174T cells to investigate their effects on gut barrier functions. Through this study, we proved that besides toxic di-terpenes, the compounds acknowledged to be non-toxic, such as licorice saponins and yuanhua flavonoids, can also take part in licorice-yuanhua-related gut damage (**Figure 1**).

## Materials and Methods

### Herbs and Chemicals

Licorice and yuanhua slices were purchased from Anhui BBCA Pharmaceutical Co., Ltd. and deposited in the Herbarium of Shaanxi University of Chinese Medicine (herbarium code: SNTCM), with voucher specimen No. DP-20110216 for yuanhua and No. GU-20100520 for licorice. The herbal samples used in this study were consistent with those in our previous research (Yu et al., 2019). Herbal slices were identified by Professor Jin-ao Duan as from roots of *Glycyrrhiza uralensis* Fish. ex DC. and flower buds of *Daphne genkwa* Siebold & Zucc., respectively. The plant names were verified using the Kew Medicinal Plant Names Service.

Chemical compounds from licorice and yuanhua including GA, GRA, GAMG, GKW, APG, HGKW, LUT, LUTG, and TLS were purchased from Nanjing Spring Autumn Biological Engineering Co., Ltd.(China), with a purity of 98%; di-terpenes of yuanhuacine (YHC) and yuanhuapine (YHP) were purchased from Chengdu Chroma-Biotechnology Co., Ltd. (China) with a purity of 99%. Sodium butyrate (SB, with a purity of 98%) and lipopolysaccharide (LPS) were purchased from Sigma-Aldrich. Antibiotics, including ampicillin, vancomycin, neomycin, and metronidazole, with purity higher than 95% were purchased from Aladdin (Shanghai).

### Herbal Extractions and Chemical Profiling

Licorice and yuanhua were extracted as we previously reported (Yu et al., 2017). Generally, licorice was decocted with water 2 times, and the supernatant was obtained and concentrated before being stored in a refrigerator at -80°C. Yuanhua was extracted similarly with 95% ethanol and vacuum-dried to acquire its total composition. The extraction percentage yields of licorice and yuanhua were 44.6% and 22.1%, respectively. Before use, the extracts were suspended with physiological saline for oral administration.

Licorice and yuanhua extracts were analyzed by ultra-performance liquid chromatography (UPLC) tandem ultraviolet detector (UV) and time-of-flight mass spectrometry (TOF-MS) (Yu et al., 2019); the detailed methods are given at the beginning of the Supplementary Information.

### Animals and Experimental Design

Seventy male mice (ICR mice, bred from Swiss mice in the Institute of Cancer Research, one of the most widely used mice in toxicology research) of 5-6 weeks old and with body weight between 18 and 22 g were fed with standard chow for one week of adaption in a temperature-controlled environment (22 ± 2°C, 55% ± 5% relative humidity) with a 12-h:12-h light-dark cycle. Mice were randomly divided into seven groups and treated with licorice and/or yuanhua extracts orally for another week. Doses for these groups were as follows: control group, physiological saline, 0.1 mL per 10 g body weight, the same among all groups); GUL and GUH group, 3.3 and 6.6 mg licorice extracts per 10 g body weight, respectively; DPL and DPH group, 0.5 and 1.0 mg yuanhua extracts per 10 g body weight, respectively; GUDPL group, 3.3 mg licorice extracts plus 0.5 mg yuanhua extracts per 10 g body weight; GUDPH group, 6.6 mg licorice extracts plus 1.0 mg yuanhua extracts per 10 g body weight.

The doses in the animal experiments were designed according to clinical doses of licorice and yuanhua. In the Chinese Pharmacopeia, the upper limit doses of licorice and yuanhua are 10 and 3 g crude herb/body/day, respectively. In this study, these upper limit doses were multiplied by 9.1 (scaling factor between human and mice) and extraction percentage yields (44.6% for licorice and 22.1% for yuanhua) to arrive at high doses of licorice (GUH) and yuanhua (DPH) for mice, in which human body weight was regarded as 60 kg. The low doses were set as one-half of the high doses.

Mice were treated once per day at about 9:00 AM. In another independent experiment, animals were simultaneously treated with antibiotics cocktail (ampicillin 1 g/L, vancomycin 0.5 g/L, neomycin 1 g/L, metronidazole 1 g/L) and herbal extracts to reduce the influence of intestinal bacteria. At the end of the experiments, mice were sacrificed, and serum and tissues from the duodenum, ileum, and colon were obtained.

All treatments were carried out with permission from the Animal Ethics Committee of Nanjing University of Chinese Medicine, according to the internationally accepted principles for laboratory animal use and care as found in the European Community guidelines (EEC Directive of 1986; 86/609/EEC). The experimental design also met the ConsEFS standards (Heinrich et al., 2018).

### Cell Culture and Treatment

The IEC-6 cells (original from rat small intestinal crypts) and LS174T cells (original from human colonic gland) were purchased from ATCC. IEC-6 cells were cultured with DMEM-high glucose medium supplemented with 10% fetal bovine serum, 1% penicillin-streptomycin solution, and 5 μg/mL insulin, while LS174T cells were cultured with RPMI 1640 medium containing 10% fetal bovine serum and 1% penicillin-streptomycin solution. All cells were incubated at 37°C, provided with 5% CO_2_ and 95% air. Before treatment, cells were passaged and planted into 6-well plates or a 100-mm dish for 12 h following treatment with drug-containing medium for another 24 h. Series concentrations of GA, GRA, GAMG, GKW, APG, HGKW, LUT, LUTG, TLS, YHC, and YHP were tested for ZO-1 and MUC-2 expressions. The doses used in cell experiments were decided according to MTT cytotoxicity assay, and doses that did not inhibit cell growth were selected. For the proteomics experiment, GA and GKW were both used at concentrations of 50 μM. LPS and SB were used as positive control, respectively. Three biological repetitions were made for the Western blot experiment and two for the proteomics experiment. After rinsing with cold phosphate buffer saline, cells were treated with lysis buffer for Western blot assay or iTRAQ-labeled proteomics assay.

### Pathological Staining

After treatment, mouse duodenum, ileum (2 cm before cecum), and colon (2 cm after cecum) tissues were kept at -80°C for Western blot analysis or in 10% neutral buffered formalin solution for hematoxylin-eosin (HE) staining. Tissue section preparation and staining were performed as previously described (Cadwell et al., 2010). All sections were imaged on the same microscope (Zeiss) with consistent acquisition parameters. HE staining sections were subjected to histopathological assessment according to a scoring system described previously (Kernbauer et al., 2014). Briefly, sections were observed blindly by two independent pathologists and were assessed for epithelial integrity (0 = no change; 0.5 = <10 epithelial cells shed per lesion; 1 = 11-20 epithelial cells shed per lesion; 2 = epithelial ulceration; 3 = epithelial ulceration with severe crypt destruction), submucosal edema (0 = no change; 0.5 = mild; 1 = moderate; 2 = profound), epithelial hyperplasia (based on percentage above height of the control, 0 = no change; 1 = <50%; 2 = 51-100%; 3 = >100%), neutrophil and mononuclear cell infiltration (0 = none; 0.5 = mild; 1 = moderate; 2 = severe). Scores for each section may be between 0-10.

### Serum Biomarker Measurement

Mouse serum was collected for quick assessment of intestinal mucosal damage, barrier function, and system inflammation. For this purpose, serum diamine oxidase activity (DAO), serum LPS concentration, and serum cytokines, including interleukin-1β (IL-1β) and interleukin-6 (IL-6), were all determined (Wolvekamp and de Bruin, 1994). DAO was measured with a biochemical analyzer by using the enzyme-linked colorimetric method; the others were measured with ELISA kits purchased from the Beijing Sino-UK Institute of Biological Technology (Beijing, China).

### Western Blot Analysis

Mouse ileum tissues were weighed and homogenized on TissueLyser with cold RIPA lysis buffer (1 mL per 100 mg tissue) for 1 min, and protein concentration was measured by Bradford assay. About 50 μg of proteins were mixed with loading buffer and subjected to sodium dodecyl sulfate-polyacrylamide gel electrophoresis. The stacking gel concentration was 4%, and resolving gels were composed of 6% and 12% gel (about 0.6 and 4.2 cm height, respectively). IEC-6 cells and LS174T cells were treated with 200 μL of loading buffer per well and directly extracted on ice for 10 min. After gel separation, proteins were transferred onto polyvinylidene fluoride membrane and blocked with 5% bovine serum albumin in TBST solution for 2 hours at room temperature then incubated overnight at 4°C in primary antibody solution. Secondary antibody incubation was performed at room temperature for 1 hour. Antibody incubations were followed by six times of 5-min washing with TBST solution. Then, the membranes were incubated in enhanced chemiluminescent solutions and visualized in a ChemiDoc XRS imaging system (Bio-Rad). In this research, mouse polyclonal antibodies to human or mouse MUC2 protein (1:5000 dilution), rabbit polyclonal antibodies to mouse or rat ZO-1 protein (1:2000 dilution) were both purchased from Abcam (USA). Polyclonal antibodies to β-tubulin protein (1:5000 dilution) and secondary antibodies (1:5000 dilution) to mouse or rabbit IgG proteins were all obtained from GeneTex (USA).

### Protein Preparation and iTRAQ Labeling

LS174T cells were treated in a 100-mm dish with GA or GKW, separately or jointly, for 24 h. Then, the cells were washed with cold phosphate buffer saline, were collected into 1.5-mL tubes, had 100 μL of SDT lysis-buffer (4% SDS, 100 mM Tris-HCl, 100 mM DTT, pH 7.6) added, and were boiled for 15 min. The samples were quantified with a BCA Protein Assay Kit. Subsequently, 100 μg of proteins per sample were subjected to filter-aided sample digestion according to a previously reported method (Wiśniewski et al., 2009). Lastly, the peptide mixtures were labeled with iTRAQ reagent according to the manufacturer’s instructions.

### Peptide Fractionation With Strong Cation Exchange (SCX) Chromatography

The iTRAQ-labeled peptides were further fractioned by the SCX method on the AKTA Purifier system (GE Healthcare). Briefly, dried peptide mixtures were reconstituted and acidified with buffer A (10 mM KH_2_PO_4_ in 25% acetonitrile, pH 3.0) and loaded onto a PolySULFOETHYL column (4.6 × 100 mm, 5 μm, 200 Å). Then, the peptides were eluted at 1 mL/min with a gradient of elution buffer B (500 mM KCl, 10 mM KH_2_PO_4_ in 25% acetonitrile, pH 3.0). Gradient: 0-22 min, 0-8% buffer B; 22-47 min, 8-52% buffer B; 47-50 min, 52-100% buffer B; 50-58 min, 100% buffer B. Ten fractions were collected during the elution period and were desalted through Empower SPE C18 Cartridges (volume 3 mL), and then these fractions were concentrated by vacuum centrifugation.

### Nano-RPLC-MS Analysis and Peptide Identification

Fractioned peptides were injected onto a nanoViper C18 trap column (100 μm × 2 cm, Thermo Scientific PepMap100) connected to a C18 analytical column (75 μm × 10 cm, 3 μm resin, Thermo Scientific Easy Column) and were eluted with buffer C (0.1% formic acid) and buffer D (0.1% formic acid and 84% acetonitrile). The flow rate was controlled to 300 nL/min by IntelliFlow software on a nano-HPLC system (Easy nLC, Proxeon Biosystems). Gradient: 0-50 min, 0-35% buffer D; 50-55 min, 35-100% buffer D; 55-60 min, 100% buffer D. Eluted peptides were detected on a tandem Q-Extractive mass spectrometer (Thermo Scientific) in positive ion mode. The top 10 precursor ions from the survey scan (300-1800 m/z) were dynamically chosen for HID fragmentation. Main parameters: automatic gain control target, 3×10^6^; maximum injection time, 10 ms; survey scan resolution, 70,000 at m/z 200; HCD spectra resolution, 17,500 at m/z 200; isolation width, 2 m/z; normalized collision energy, 30 eV; underfill ratio, 0.1%.

MS/MS spectra were searched by the MASCOT engine (version 2.2, Matrix Science, UK) embedded in Proteome Discoverer 1.4 software. The main parameters were as follows: enzyme, trypsin; max missed cleavages, 2; fixed modifications, Carbamidomethyl (C) and iTRAQ4/8plex (N-term and K); variable modifications, oxidation (M) and iTRAQ4/8plex (Y); peptide mass tolerance, 20 ppm; fragment mass tolerance, 0.1 Da; Database pattern, Decoy; peptide FDR threshold, 0.01. The database used was downloaded from the UniProt website (uniprot_Human_159691_20170809.fasta).

### KEGG Pathway Annotation and Enrichment Analysis

The FASTA protein sequences of differentially changed proteins were blasted against the online Kyoto Encyclopedia of Genes and Genomes database to retrieve KOs, which were subsequently mapped into pathways in KEGG (Moriya et al., 2007). KEGG pathway enrichment analyses were then applied to discover internal relationships between differentially expressed proteins based on Fisher’s exact test, considering the whole quantified protein annotations as the background dataset.

### Statistical Analysis

Serum biomarker levels were compared by one-way analysis of variance followed by Dunnett’s t-test (SPSS 11.5); histopathological data were analyzed by the non-parameter Mann-Whitney U test; Western blot analysis data were compared with the Student’s t-test or Mann-Whitney U test. Significant thresholds of comparisons for these data were set at 0.05. In the iTRAQ-labeled proteomics study, protein ratios were calculated based on the median of all unique peptides belonging to that protein, and ratios were normalized by the median of all proteins detected. Ratios between 0.83 and 1.2 with a significant A value lower than 0.05 were considered as differentially expressed proteins.

## Results

### Chemical Profile of the Licorice-Yuanhua Herbal Pair

In this study, licorice and yuanhua were extracted with water and ethanol, respectively. Then, the licorice-yuanhua herbal pair was made by mixing their extracts directly to avoid the dissolution-promoting effect of licorice saponins on the compounds of yuanhua (Chen et al., 2012).

**Figure 2:**
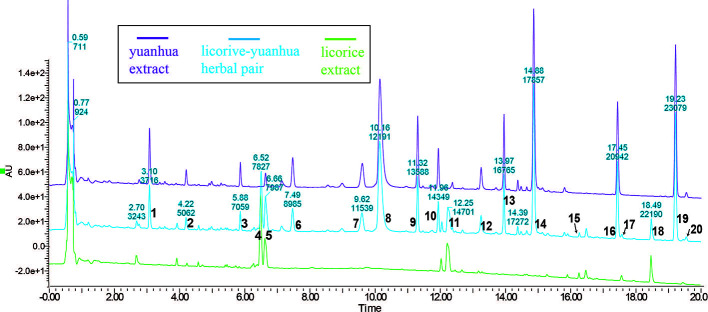
UPLC profiles of licorice, yuanhua, and the licorice-yuanhua herbal pair. Wavelength of UV-detector was set at 254 nm, and most peaks appear within 20 mins. These compound peaks were identified by the UPLC-TOF-MS method: 1, phenolic acid (possibly Neochlorogenic acid, 3-*O*-Caffeoylquinic acid or 5-*O*-Caffeolyquinic acid); 2, Cinnamic acid; 3, Luteolin-5-*O*-glucoside; 4, Liquiritin/Isoliquiritin; 5, overlapping of Liquiritin/Isoliquiritin apioside (from licorice) and Apigenin 6-*C*-glucoside-8-*C*-arabinoside (from yuanhua); 6, flavonoid glycoside (possibly Apigenin 5-β-D-glucopyranoside, Apigenin-6-*C*-glucoside, or Apigenin 7-*O*-β-D-glucopyranoside); 7, Leucanthoside or 3’-Hydroxygenkwanin 5-*O*-β-D-glucoside; 8, mix of Magnesium pheophytin and Apigenin-7-*O*-β-D glucuronoside; 9, flavonoid glycoside (possibly Apigenin 7-rhamnoglucoside, Kaempferitrin, 4’,5-dihydroxy-7-methoxy-5-(*O*-xyloglucoside)-flavone, Genkwanin 5-*O*-β-D-primeveroside, or Yuenkanin); 10, Genkwanin 5-*O*-β-D-glucoside; 11, flavonoid (possibly Liquiritigenin, Isoliquiritigenin, or Pinocembrin); 12, Luteoline; 13, Tiliroside; 14, Apigenin; 15, 22-Acetoxyl-glycyrrhizin; 16, Hydroxygenkwanin; 17, triterpenoid saponin (possibly Yunganoside K2, Licorice saponin G2, or 22-Hydroxy-glycyrrhizin); 18, Glycyrrhizin; 19, Genkwanin; 20, triterpenoid saponin (possibly Licorice saponin H2/K2, 18α-Glycyrrhizin, Uralsaponin B, or Yunganoside L2/J2). The UPLC-TOF-MS profiles of licorice and yuanhua are given in the supplementary information, and the complete lists of identification compounds are also provided (**Supplementary Figures S1** and **S2** and **Tables S1** and **S2**).

To characterize the material basis of the licorice-yuanhua herbal pair, we first compared the UPLC-UV profiles of licorice, yuanhua, and their combination; then, UPLC-TOF-MS analysis was conducted for compound identification. In UPLC analysis, most of the compounds were eluted within 20 min (**Figure 2**). Each compound peak in the herbal pair comes from licorice or yuanhua, and yuanhua contributed more compounds than licorice. These compound peaks were simultaneously analyzed by high-resolution mass spectrometry and searched against a homemade compound database (Yu et al., 2019). In total, 20 peaks in the UPLC profile were identified, among which 14 are flavonoids (compounds 3-14, 16, and 19) and four are saponins (compounds 15, 17, 18, and 20).

The full UPLC-TOF-MS profiles and lists of compounds identified in licorice and yuanhua are given in the supplementary information (**Supplementary Figures S1** and **S2** and **Tables S1** and **S2**), in which yuanhua di-terpenes were also detected, including YHC and YHP, but with very low detected responses.

### The Licorice-Yuanhua Herbal Pair Induces Ileum Mucosal Inflammation Related With Weakened Barrier Functions and Bacterial Infections

**Figure 3:**
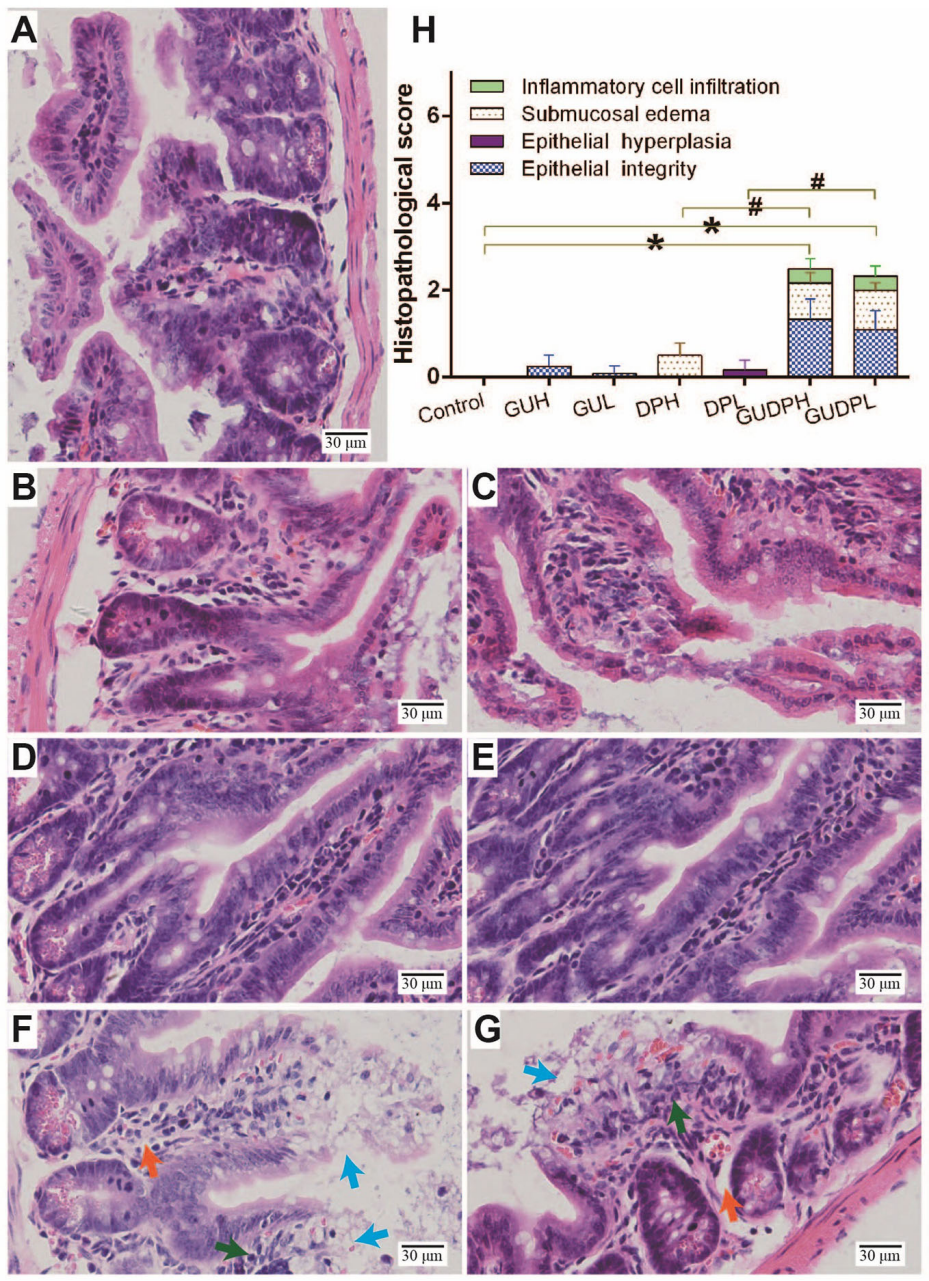
Histopathological staining and evaluation of mouse ileum. HE staining of mouse ileum tissues in each group: **(A)** Control, **(B)** GUH, **(C)** GUL, **(D)** DPH, **(E)** DPL, **(F)** GUDPH, and **(G)** GUDPL groups. Blue arrows are signs of epithelial integrity loss, orange arrows are signs of submucosal edema, and green arrows are signs of inflammation cell infiltration. **(H)** Bar chart of histopathological scores of ileum tissues in each group. *P < 0.05 compared with control group, ^#^*P* < 0.05 compared between herbal pair and single herb, as indicated by connecting lines.

To assess whether licorice-yuanhua could induce gut injury, mice were exposed with licorice extract and yuanhua extract separately or jointly for one week, and the mouse guts were then analyzed through histopathologic analysis. Mouse duodenum, ileum, and colon tissues were observed and scored on the aspects of inflammation, edema, epithelial hyperplasia, and epithelial integrity loss. The results (**Supplementary Figure S3**) show that mouse duodenum was slightly damaged by yuanhua with or without licorice combination, verifying the toxicity of yuanhua on the gastrointestinal system. Licorice had no effect on mouse duodenum and did not influence the effect of yuanhua. As for the colon, no damage was observed in any group, and no interaction was found between licorice and yuanhua (**Supplementary Figure S4**). However, in mouse ileum, mild damage was found in jointly treated groups (GUDPH and GUDPL) rather than in yuanhua groups, mainly in terms of epithelial integrity loss, edema, and inflammation (**Figure 3**). The accumulative histopathology score in the GUDPH and GUDPL groups was significantly elevated compared with control or yuanhua groups (DPH and DPL). Here, in ileum, licorice or yuanhua did not lead to obvious injury separately, since damage could only be induced by their combination. The results provide evidence for the common sense among TCM doctors that the licorice-yuanhua combination can induce toxicity. Nevertheless, the problems of why and how ileum is damaged remain to be resolved.

**Figure 4:**
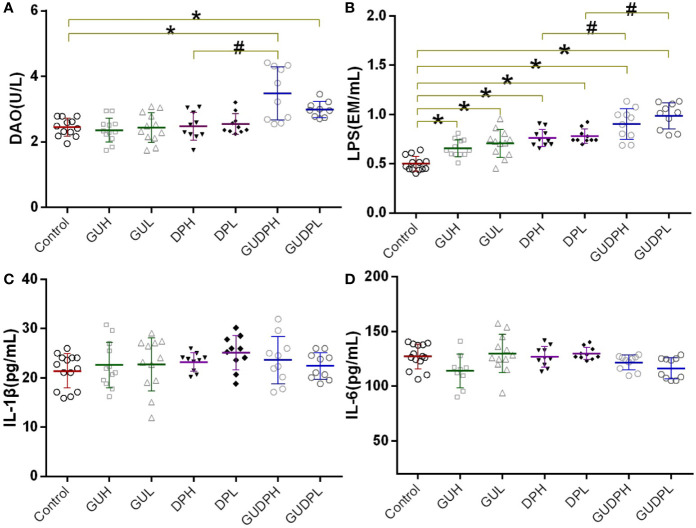
Mouse serum biomarkers related to mucosal tissue and barrier function damage. **(A)** Serum DAO levels of mice. **(B)** Serum LPS levels of mice. **(C, D)** Serum cytokine levels of mice. *P < 0.05 compared with control group, ^#^P < 0.05 compared between GUDPH and DPH groups or between GUDPL and DPL groups, as indicated by connecting lines. GUH, licorice high dose; GUL, licorice low dose; DPH, yuanhua high dose; DPL, yuanhua low dose; GUDPH, herbal pair high dose; GUDPL, herbal pair low dose.

Since ileum injury induced by the licorice-yuanhua herbal pair is chiefly on gut mucosa inflammation and integrity loss, biomarkers are employed to prove the occurrence of these types of damage. Serum DAO activity and LPS level have been recognized as sensitive indicators for gut mucosa damage and gut barrier functions, respectively (Giménez-Gómez et al., 2019). Serum IL-1β and IL-6 levels have been used as indicators of system inflammation. These biomarkers were determined as shown in **Figure 4**. DAO levels in the GUDPH group are significantly higher than in both the control and DPH groups; DAO levels in the GUDPL group are also higher than in the control group. LPS levels in all of the treated groups are significantly higher than in the control group, and LPS levels in the GUDPH and GUDPL groups are further increased compared with the DPH or DPL groups. Mouse serum IL-1β and IL-6 levels are not significantly changed in any treatment group. These results suggest that the licorice-yuanhua herbal pair can significantly damage intestinal mucosa and weaken gut barrier functions more strongly than can licorice or yuanhua alone. Although licorice and yuanhua did not damage mouse ileum, they both weakened gut barrier functions and exacerbated this effect when they were used jointly. Considering the histopathological staining results, we can conclude that injuries induced by the licorice-yuanhua herbal pair mainly happened and exacerbated in mouse ileum. Notably, injuries in mouse gut were relatively mild because system inflammation levels were not influenced.

Mouse ileums were then analyzed by Western blot to determine inflammation level and barrier functions by measuring tissue cytokines (IL-1β and IL-6) and tight junction protein (ZO-1) expression as well as mucus protein (MUC-2) expression. ZO-1 protein is one of the most important elements in tight junction assembly of mucosal epithelial cells, and MUC-2 protein is the main component of mucus secreted by goblet cells (Obert et al., 2017). ZO-1 and MUC-2 proteins are recognized as markers of epithelial and mucous barrier functions, respectively (Sicard et al., 2017).

**Figure 5:**
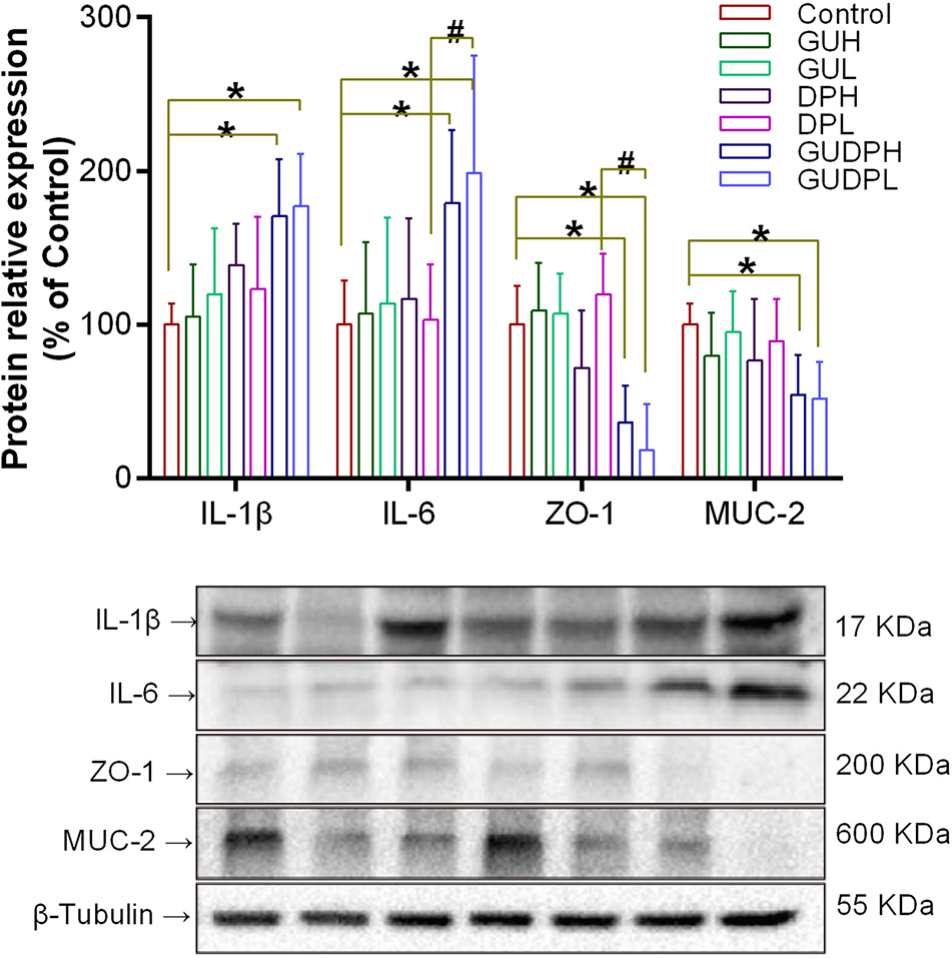
Protein expressions related to inflammation and barrier functions in mouse ileum. Upper panel: bar chart of IL-1β, IL-6, ZO-1, and MUC-2 expressions. Lower panel: blotting images of these proteins with β-tubulin used as internal control. *P < 0.05 compared with control group, ^#^P < 0.05 compared between GUDPH and DPH groups or between GUDPL and DPL groups, as indicated by connecting lines. GUH, licorice high dose; GUL, licorice low dose; DPH, yuanhua high dose; DPL, yuanhua low dose; GUDPH, herbal pair high dose; GUDPL, herbal pair low dose.

The results (**Figure 5**) show that the expression levels of ileum IL-1β and IL-6 in the GUDPH and GUDPL groups were both significantly increased compared to the control group and that IL-6 levels in the GUDPL group were also higher than in the DPL group. The expression levels of ZO-1 protein in ileum were significantly decreased in the GUDPH and GUDPL groups compared with the control group, and ZO-1 expression levels in the GUDPL group were significantly lower than in the DPL group. Similarly, MUC-2 protein expressions in ileum are also significantly decreased in the GUDPH and GUDPL groups compared with in the control group. The interleukin levels and ZO-1 and MUC-2 expressions were not changed in the licorice- and yuanhua-only groups. These results confirm that the licorice-yuanhua herbal pair leads to mouse ileum inflammation and damages its barrier functions through downregulating proteins involved in tight junction and mucus generation.

Considering that licorice is well-known as a non-toxic herb and that neither licorice nor yuanhua exerts obvious toxic effects to mouse ileum at the given doses, we believe that the ileum damage induced by the licorice-yuanhua herbal pair was not caused by a direct overlap of cytotoxicity of the two herbs, although yuanhua has some toxic compounds within it. As we have shown, both licorice and yuanhua can weaken mouse gut barrier function, leading to higher LPS levels in mouse blood. Although licorice or yuanhua rarely influenced ZO-1 and MUC-2 expressions, their combination significantly reduced ZO-1 and MUC-2 expressions, which was well consistent with the serum LPS levels.

Gut epithelial tight junction and mucous secretion are both important parts of gut barrier function, isolating pathogens from gut epithelial cells and preventing infections and inflammations (Sicard et al., 2017). Given this information, a conclusion could be made that licorice-yuanhua-induced injuries in mouse ileum may be related to weakened gut barrier functions and subsequent bacterial infections.

### Antibiotics Abolish Ileum Injuries Induced by the Licorice-Yuanhua Herbal Pair

**Figure 6:**
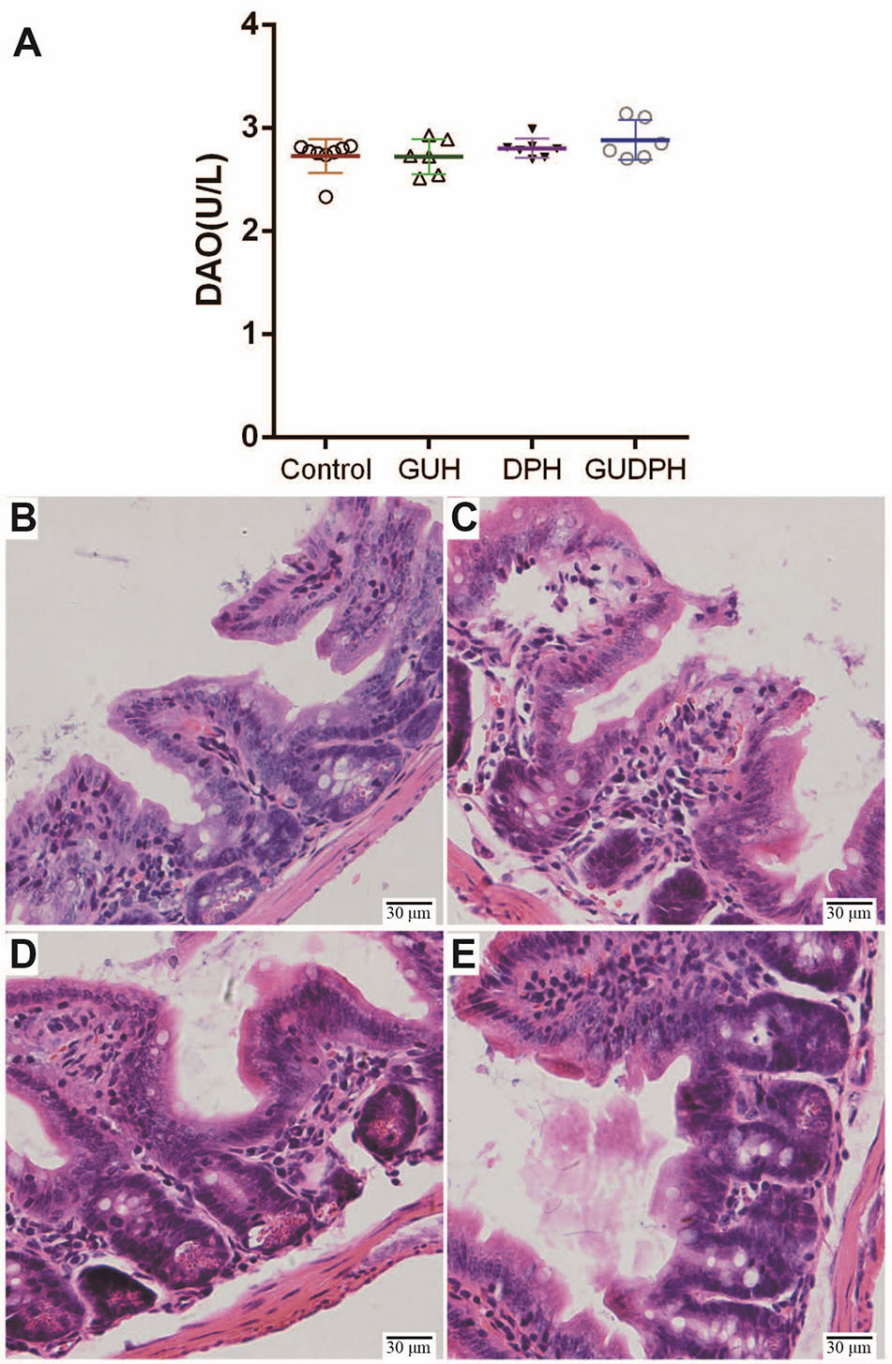
Serum DAO levels and HE staining of ileum tissues in mice treated simultaneously with antibiotics and herbal extracts. **(A)** Mouse serum DAO levels in each group. **(B)** HE staining of mouse ileum tissue slices in Control group. **(C)** HE staining of mouse ileum tissue slices in GUH group. **(D)** HE staining of mouse ileum tissue slices in DPH group. **(E)** HE staining of mouse ileum tissue slices in GUDPH group.

To investigate the roles of gut bacteria in licorice-yuanhua-induced ileum injuries, four groups of mice were treated with antibiotics during licorice and/or yuanhua treatment. The serum DAO levels of the mice were measured, and their ileum tissues were analyzed with HE staining (**Figure 6**). No significant difference was found in the DAO levels of these four groups, and HE staining showed that occasional damage existed in mouse ileum, but no obvious differences were found among these groups. The occasional damage may have been related to the antibiotics themselves, because this phenomenon was also found in the control group.

That is, antibiotics abolish ileum injuries induced by the licorice-yuanhua herbal pair. These results lead us to believe that licorice-yuanhua-induced mucosal damage in mouse ileum is related to weakened gut barrier functions and subsequent bacterial infections.

### The iTRAQ-Labeled Proteomic Study Indicated That GA and GKW Synergistically Regulate Cellular Tight Junction Pathways and Pathogen Infection Pathways in LS174T Cells

In the next step of this study, we investigate which compounds in the licorice-yuanhua herbal pair may be responsible for weakened gut barrier functions and consider whether it is possible that both licorice and yuanhua contain gut barrier-weakening compounds and that these compounds jointly reduce gut barrier functions. In licorice, GA is recognized as the main active compound responsible for its traditional therapeutic efficacy, while in yuanhua, di-terpenes and flavonoids are both important compounds for its efficacy, and di-terpenes are also responsible for the toxicity of yuanhua. Given that the ileum injuries may not be caused directly by toxic di-terpenes and that licorice is a detoxifying herb, we believe that di-terpenes may not be the key compounds in this study.

As a result, we assumed that licorice saponins and yuanhua flavonoids may weaken gut barrier functions. To further prove this possibility, GA and GKW were incubated separately or jointly with LS174T cells, and proteomes of cells were analyzed by iTRAQ-labeled proteomic technology.

After protein extraction, digestion, labeling, pooling, and SCX fractionation, samples were subjected to one-hour elution schedules on nano-RPLC systems, and, in total, 33,455 peptides were detected by tandem mass spectrometer and were identified with quantity information. Among these peptides, 30,225 were determined to be unique peptides and then grouped into 5295 proteins. The molecular weights of these identified proteins range from 1.9 kDa to 838 kDa (**Supplementary Figure S5**), showing the powerful adaptability of the iTRAQ-labeled proteomic technology. Of the 5,295 proteins, the vast majority were kept unchanged (**Supplementary Figure S6**); only 228 proteins (4.3%) were upregulated and 353 proteins (6.7%) downregulated by GA, GKW, or GA-GKW combination (GAGKW) treatment.

**Figure 7:**
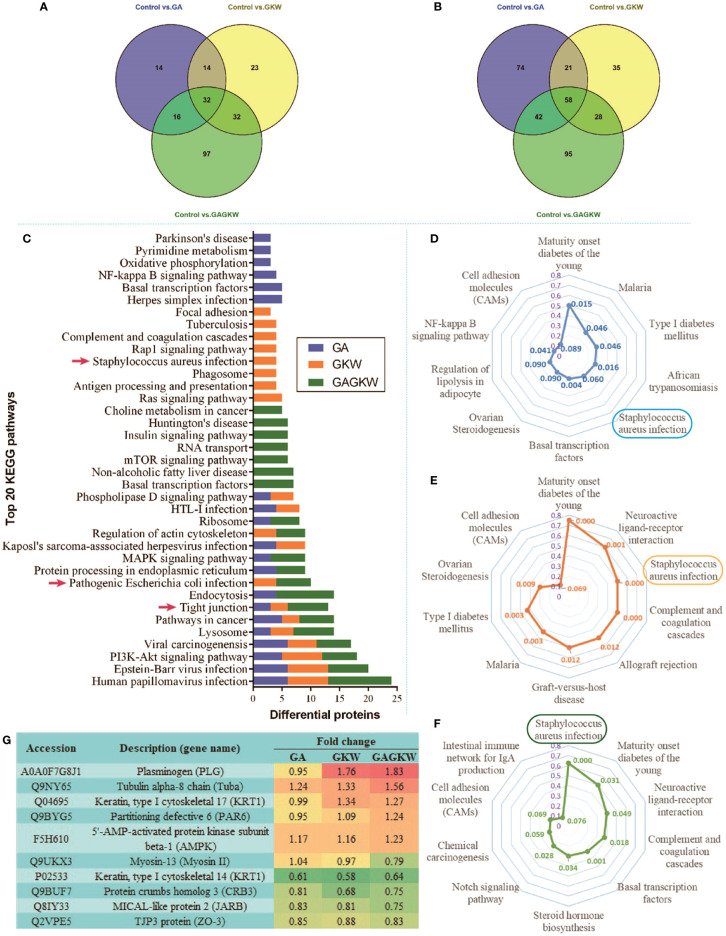
iTRAQ-labeled proteomic study in LS174T cells treated by GA, GKW, and GAGKW. **(A)** Venn diagram of upregulated proteins treated by GA, GKW, and GAGKW. **(B)** Venn diagram of downregulated proteins treated by GA, GKW, and GAGKW. **(C)** Top 20 KEGG pathways regulated by GA, GKW, and GAGKW. Red arrows indicate pathogen infection pathways and the tight junction pathway. **(D–F)** Radar maps of KEGG pathway enrichment for GA, GKW, and GAGKW treatment, respectively. The maximum number for enrichment factor (purple axis) is set at 0.8, and p-values for each pathway are given beside pathway nodes. **(G)** Fold changes of proteins involved in the *Staphylococcus aureus* infection pathway and tight junction pathway.

Of the upregulated proteins, 76, 101, and 177 proteins were caused by GA, GKW, and GAGKW treatment, respectively. Of the downregulated proteins, 195, 142, and 223 proteins were caused by GA, GKW, and GAGKW treatment, respectively. Additionally, 94 (41% of 228) upregulated and 149 (42% of 353) downregulated proteins were shared among groups, and 97 and 95 extra proteins were uniquely upregulated or downregulated by GAGKW treatment, respectively (**Figures 7A, B**). These numbers collectively suggest that the regulation effects of GA and GKW in LS174T cells partially overlap with each other and that the GA-GKW combination generates new regulation profiles that differ from GA or GKW and also differ from the union of GA and GKW.

**Figure 8:**
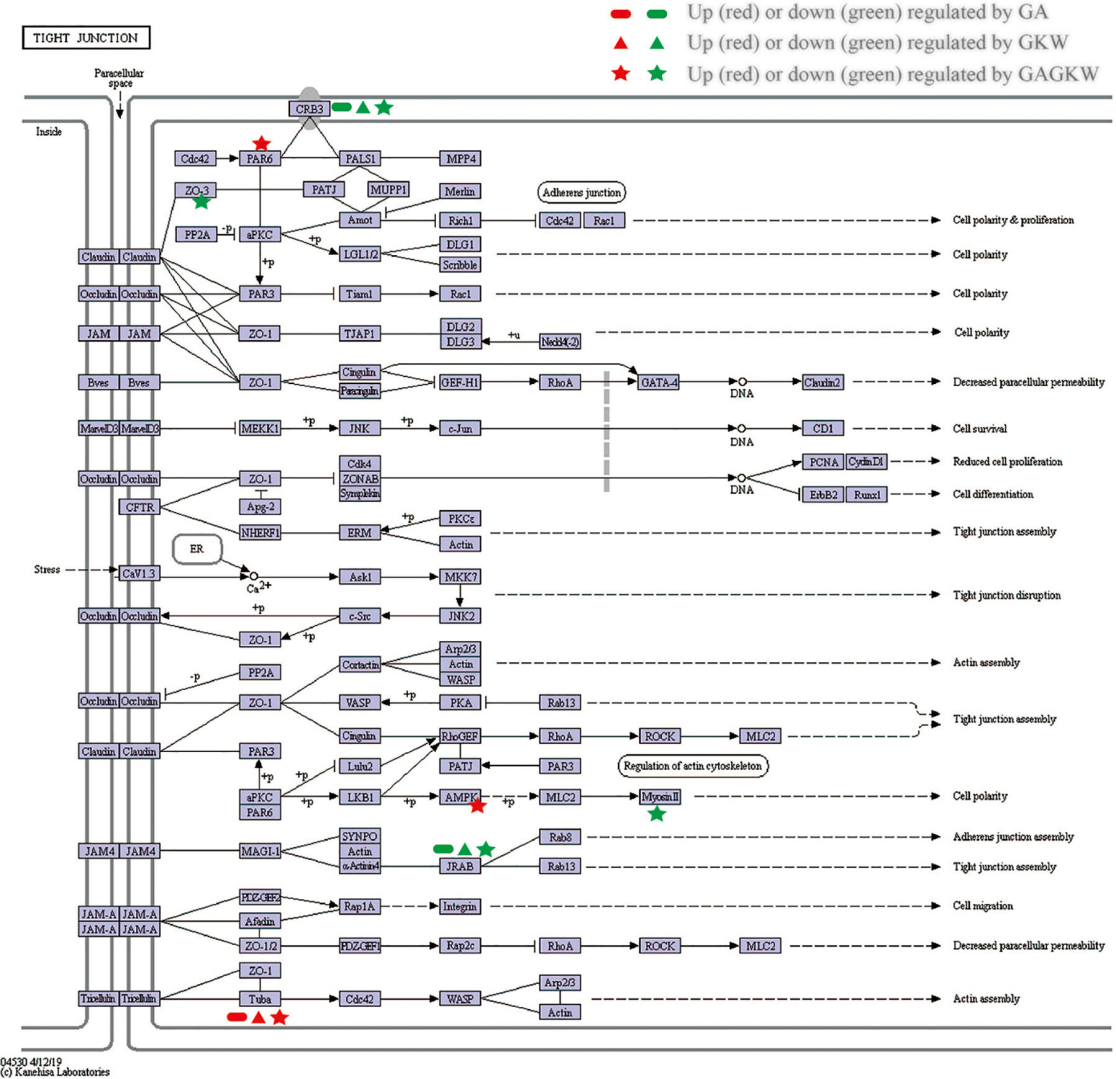
KEGG Pathway map for tight junction. Regulated genes are signed with indicated characters and colors.

The upregulated and downregulated proteins were next mapped into KEGG pathways; the top 20 pathways of each treatment group are shown in **Figure 7C**. It can be concluded that GA, GKW, and also GAGKW all have similar regulation profiles in pathogen infection (such as virus infections and bacterial infections), cancer, and tight junction pathways. For the tight junction pathway, there are 3, 3, and 7 regulated proteins engaged in GA, GKW, and GAGKW treatment, respectively. Pathway enrichment analysis (**Figures 7D–F**) shows that pathogenic bacteria infection pathways are regulated by both GA and GKW, and the enrichment factor of the *Staphylococcus aureus* infection pathway is the highest in GAGKW treatment group. The expression patterns of proteins involved in the tight junction pathway (related genes: Tuba, PAR6, AMPK, Myosin II, CRB3, JARB, and ZO-3) and *Staphylococcus aureus* infection pathway (related genes: PLG and KRT1) also show synergistic regulation effects of GA and GKW (**Figure 7G**). In the tight junction pathway, GA, GKW, and GAGKW all downregulate protein crumbs homolog 3 and MICAL-like protein 2, and all upregulate tubulin alpha-8 chain. The regulation effect of GAGKW is stronger than that of GA or GKW. More importantly, GAGKW regulates more proteins than GA or KGW, such as TJP3 protein and Myosin-13 protein (**Figure 8**); these regulated proteins are related to downregulated functions of cell polarity and tight junction assembly, and this result was further validated in the Western blot experiments, as detailed below.

### Besides Di-Terpenes, Saponins and Flavonoids in The Licorice-Yuanhua Herbal Pair Dose/Structure-Dependently Decreased ZO-1 and MUC-2 Expressions

Based on the above results, we began to verify the regulation effects of licorice saponins and yuanhua flavonoids on mucosal epithelial tight junction, as well as mucus secretion. According to the UPLC-MS/MS experiment, we selected representative saponins and flavonoids in licorice and yuanhua and incubated these compounds with IEC-6 cells and LS174T cells, respectively, to study the regulation effects of these compounds on ZO-1 and MUC-2 expressions.

Because GA can be metabolized as GAMG and GRA in the gut lumen, these compounds were all selected. Yuanhua flavonoids of GKW, HGKW, APG, LUT, LUTG, and TLS were all employed in this study. Additionally, yuanhua di-terpenes like YHC and YHP were also studied. The chemical structures of these compounds are shown in **Supplementary Figure S7**. In the cell experiments, LPS and SB were used as positive control, respectively, for IEC-6 cells and LS174T cells.

**Figure 9:**
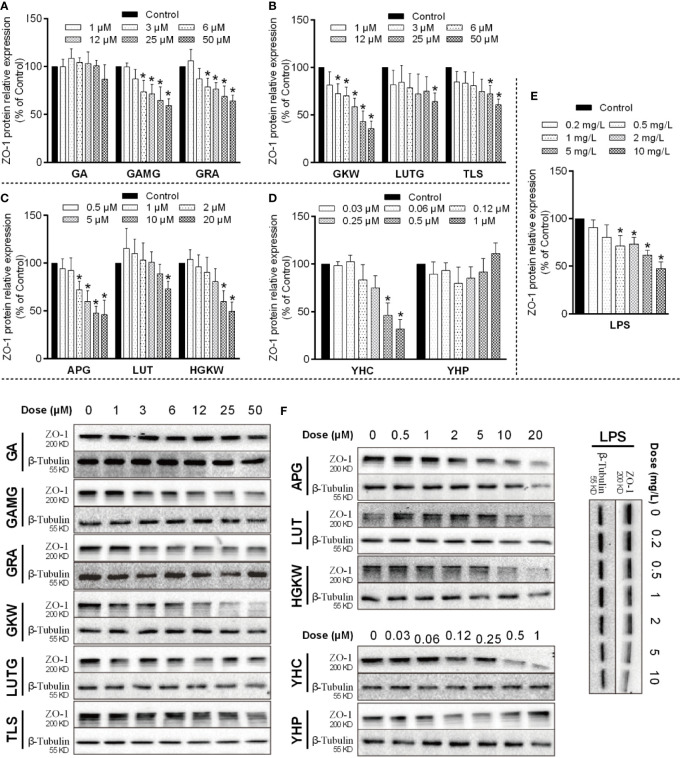
ZO-1 expressions influenced by saponins, flavonoids, and di-terpenes in IEC-6 cells. **(A–E)** Bar chart of ZO-1 expressions in IEC-6 cells treated with the indicated compounds. *P < 0.05 compared with control group. **(F)** Blotting images of ZO-1 expressions in IEC-6 cells treated with the indicated compounds. LPS is used as positive material, and β-tubulin is used as internal control.

IEC-6 cells originate from epithelia of rat intestinal crypt and were used to study barrier functions of mucosa epithelia. In IEC-6 cells, ZO-1 protein expression was changed by both saponins and flavonoids as well as di-terpenes (**Figure 9**). The positive material LPS dose-dependently reduced ZO-1 expression at doses between 1 and 10 mg/L. GAMG, GRA, GKW, TLS, APG, HGKW, and YHC all reduced ZO-1 protein expression in a dose-dependent way. GA and YHP did not exert an obvious effect on ZO-1 expression, and LUT and LUTG only had effects at the highest dose. Among these compounds, structure–activity relationships are clear: for licorice saponins and yuanhua flavonoids, glycosyl derivatives weaken the activity when comparing GA with GRA or comparing LUT with LUTG. For flavonoid aglycones, a 3’-OH substituent also weakens the activity when comparing GKW with HGKW or comparing APG with LUT. For YHC and YHP, substituents also influence the activity.

**Figure 10:**
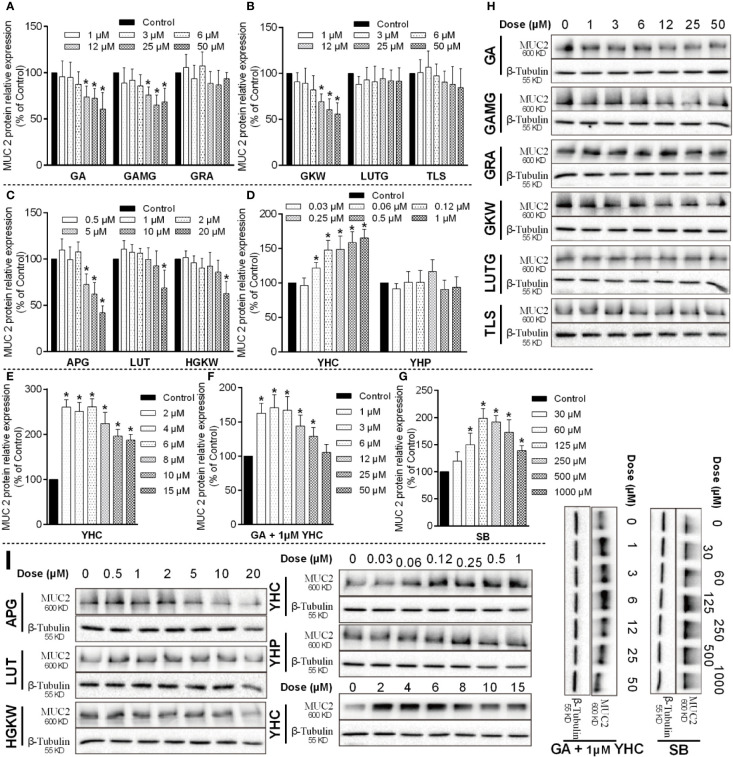
MUC-2 protein expressions influenced by saponins, flavonoids, and di-terpenes in LS174T cells. **(A–G)** Bar chart of MUC-2 expressions in LS174T cells treated by the indicated compounds. *P < 0.05 compared with control group. **(H, I)** Blotting images of MUC-2 expressions in LS174T cells treated by the indicated compounds. LPS is used as positive material, and β-tubulin is used as internal control.

LS174T cells originating from human colorectal adenocarcinoma were used to study the mucus secretion function of goblet cells. In LS174T cells, MUC-2 expression was also influenced by saponins, flavonoids, and di-terpenes (**Figure 10**). Positive material SB significantly increased MUC-2 protein expression at concentrations higher than 30 μM, and its efficacy began to fall after 125 μM concentration (Jung et al., 2015). GA, GAMG, GKW, and APG all dose-dependently decreased MUC-2 expression in L174T cells, while LUT and HGKW had much lower activities. YHC sharply increased MUC-2 expression; its activity rose between 0.06 and 1 μM and fell between 2 and 15 μM. YHP had little effect on MUC-2 expression. Similar to IEC-6 cells, these compounds also exhibit obvious structure–activity relationships, but differently, for GA and GRA, glycosyl derivatives enhance the activity rather than reducing it.

Since YHC in yuanhua upregulated MUC-2 expression, we additionally tested the activity of GA in the presence of 1 μM YHC. The results show that GA attenuated the effects of YHC and abolish the effect of YHC at 50 μM concentration. Actually, in the licorice-yuanhua herbal pair used in this study, the ratio of their concentration (GA/YHC) is about 1000:1, far higher than the 50:1 used in the LS174T cells.

The results from the cell experiments suggest that saponins and flavonoids in the licorice-yuanhua herbal pair were both responsible for weakened gut barrier functions related to mucosa epithelial tight junction and mucus secretion. The overlap in their activities may generate a synergistic effect between licorice and yuanhua, causing this herbal pair to induce gut inflammation and bacterial infection, as was shown in the animal experiments.

In summary, these results again confirm that saponins and flavonoids in the licorice-yuanhua herbal pair can both weaken gut barrier functions and may promote pathogen infections that subsequently cause gut injuries.

## Discussion

This study focuses on the intestinal toxicity of the licorice-yuanhua herbal pair, one of the incompatible herbal pairs in the “eighteen incompatibilities” theory in TCM. It was previously well known that yuanhua causes some irritation to the intestinal tract but is relatively safe at common doses in TCM clinics (Chen et al., 2013b; Qiao et al., 2014). However, TCM doctors believe that once combined with licorice, the most frequently used herb in TCM prescriptions, it will lead to enhanced toxicity to the body (Chen et al., 2019). The results of this study confirm this notion, with slight damage found in duodenum in yuanhua-treated mice and enhanced damage found in ileum in licorice-yuanhua-treated mice. The rest of our work aimed at uncovering the mechanisms and material basis of the enhanced damage in mouse ileum, and we found that the ileum damage is related to weakened gut barrier function and bacterial infections. Further, the iTRAQ-labeled proteomic study suggested that GA and GKW have regulatory effects on the tight junction pathway and bacterial infection pathways in gut mucosal cells. After structure–activity experiments, we further confirmed that both saponins and flavonoids can dose/structure-dependently reduce ZO-1 and MUC-2 expressions in gut mucosal epithelial cells, the two important elements in epithelial tight junction assembly and mucus secretion.

In this study, the data and evidence suggest that saponins and flavonoids, compounds commonly known to be non-toxic and safe for the body, can also take part in gut barrier damage and promote gut inflammation and infection, at least in the situation of licorice-yuanhua combination. The results of this study provide important evidence and insights for better understanding of the incompatibility mechanisms of the licorice-yuanhua herbal pair. Previous reports did not take notice of the different parts of the gut lumen, and as a result, they did not get detailed toxic information about the gut, only indicating that the gut may be stimulated and metabolic functions may be impaired (Chen et al., 2015; Chen et al., 2016). Thus, this study is a good complement to previous work.

Notably, we find that yuanhua treatment leads to mild damage to the duodenum rather than the ileum of mice. This may be because duodenum contacts toxic yuanhua di-terpenes (such as YHC) earlier than ileum, and the concentrations of di-terpenes are diluted along the gut lumen during the absorption process (Chen, 2014). But even so, mouse ileum was obviously injured by the licorice-yuanhua herbal pair. One of the reasons for this may be that the bacterial content in ileum is far higher than in duodenum, so bacterial infections happen much more easily in ileum (Donaldson et al., 2016). In mouse colon, however, little injury was found, in clear contrast to ileum and duodenum. There are two possible reasons for this: one is that in the colon, a large amount of mucus is secreted to protect the mucosal epithelia against bacteria, and it is harder to break the mucus barrier than in the ileum (Atuma et al., 2001). In HE staining of slices of colon tissue, a large amount of goblet cells, which generate mucus, were found to exist (**Supplementary Figure S4**). Another reason is that saponins and flavonoids are mainly absorbed in small intestine, especially in ileum, and these compounds will be enriched in small intestine due to “hepato-enteral circulation” rather than enriched in large intestine (Roberts et al., 2002). All of this information again suggests that di-terpenes may not be the key compounds in licorice-yuanhua-induced ileum injuries.

The extraction methods of the herbs were designed according to the forms of application of licorice and yuanhua. In TCM prescriptions, licorice is most frequently used in decoctions prepared by boiling in water. In this form, a large amount of licorice saponins can be extracted, which are the commonly known active components of licorice. Yuanhua is mostly used by water extraction (in TCM decoctions), ethanol extraction (in TCM tinctures such as the Yuan-Hua-Tu-Si-Zi Tincture from the ancient book “*Pu Ji Fang*”), or just in powder forms (such as the Shi-Zao Decoction from the ancient book “*Shang Han Lun*”). In this study, we extracted yuanhua with ethanol for two reasons: on the one hand, we aimed at obtaining the full components of yuanhua, both those with weak and with strong polarity; on the other hand, the water extraction method rarely gains flavonoid aglycones and di-terpenes from yuanhua, which are important compounds for yuanhua efficacy and toxicity.

The licorice-yuanhua herbal pair was made by mixing the single extracts directly rather than extracting them simultaneously, based on the consideration that when licorice and yuanhua were extracted together, the dissolution rate of di-terpenes and flavonoids in yuanhua will be significantly increased by licorice saponins (Chen, 2014), which would complicate the mechanisms of toxicity of licorice-yuanhua, as well as making it hard to compare the herbal pair with single licorice and yuanhua. Thus, in this study, we extracted licorice and yuanhua separately to avoid in-solution interactions between saponins and other compounds and to simplify the study and make it easier to compare the herbal pair with single licorice and yuanhua.

In the UPLC profile of the licorice-yuanhua herbal pair, most of the compound peaks are contributed by yuanhua (**Figure 1**) and the main compounds in the licorice-yuanhua herbal pair are saponins and flavonoids, so we chose these compounds for the cell experiments. In licorice extract, GA is the main small molecule in licorice extract, although a low content of flavonoids also exists (much lower than of GKW and APG in the licorice-yuanhua herbal pair); thus, in this study, licorice flavonoids were not yet taken into consideration. Additionally, a large amount of polysaccharides, proteins, and fatty acids exist in licorice and yuanhua extracts, which were also not considered in the cell experiments. These compounds need to be investigated in further studies.

In the cell experiments, compound doses were determined with MTT assays, and we found that APG, LUT, and HGKW inhibited cell growth at doses higher than 25 μM, while GKW, LUTG, and TLS did not inhibit cell growth, even at a 50-μM concentration. In the animal experiments, mice in the high dose herbal pair groups equivalently took into about 1 mg GA per mouse and 10 μg GKW, 15 μg APG, and 1 μg YHC. However, the concentration of these compounds in ileum mucosa needs to be clarified in future studies.

In the LS174T cells, we found downregulation effects of GA and GKW on MUC-2 expressions, but this result was not reproduced in iTRAQ-labeled proteomics experiments. This may be caused by the instabilities of MUC-2 protein in the SCX fractionation procedure. It has been reported in the literature that MUC-2 protein undergoes autocatalytic cleavage in low pH solution (Khatri et al., 1998; Lidell et al., 2003), and the elution buffer used in the SCX fractionation procedure, was acidic, with a pH of 3.0. Our study suggests that Western blot technology and proteomic technology should be used in a complementary way.

In the iTRAQ-labeled proteomics experiments, proteins involved in the tight junction pathway and the bacterial infection pathway were regulated by GA and GKW in a synergistic way. CRB3 protein is widely expressed in gut mucosa epithelial cells and is involved in the establishment of cell polarity and regulates the morphogenesis of tight junctions (Madigan et al., 2019). MICAL Like 2 (JARB) protein is also involved in regulating actin cytoskeleton reorganization and cell adhesion molecules (Sakane et al., 2012). Downregulation of CRB3 and JRAB will hinder tight junction assembly and weaken gut barrier functions. Consistent with JARB upregulation, cytoskeleton protein tubulin alpha-8 is upregulated by GA and GKW, suggesting reduced reorganization of cell cytoskeleton (Glotfelty et al., 2014). TJP-3 (ZO-3) protein links tight junction transmembrane proteins to the actin cytoskeleton, and it is necessary for epithelial polarization and barrier formation. TJP-2 and TJP-1 are similar to TJP-3. In this study, licorice saponins and yuanhua flavonoids downregulated ZO-1 proteins in IEC-6 cells while downregulating ZO-3 proteins in LS174T cells; this difference may be caused by distinctions between the two type of cells. Myosin-13 (Myosin II) gene is responsible for ATP hydrolysis and actin binding, and downregulating Myosin II gene by GAGKW treatment will reduce the efficacy of cell energy transduction and slow down the cell polarity process (Cunningham and Turner, 2012).

Plasminogen (PLG) and keratin (KRT1) proteins are expressed in epithelial cells and are important in bacterial infection pathways (Baker et al., 2019; Kochi et al., 2019). Keratin proteins are distributed on the epithelial cell surface and provide anchor points for bacteria adhesion and colonization (Martinez-Rossi et al., 2017). Plasminogen is expressed in cytoplasm and can be activated by staphylokinases of *Staphylococcus aureus* following inhibition of cell complement activation and degradation of immune proteins like IgG and C3b (Rooijakkers et al., 2005), so upregulation of the two proteins may exacerbate pathogen infections like *Staphylococcus aureus* and *Escherichia coli*. Further studies should be carried out to confirm the regulation effects of GA and GKW on pathogen infections.

In conclusion, the findings in our study not only uncover the mechanisms and chemical basis of toxic effects induced by the licorice-yuanhua herbal pair but also show a phenomenon in which saponins and flavonoids, commonly known to be non-toxic compounds, can take part in gut injuries induced by herbal–herbal interactions.

## Data Availability Statement

The mass spectrometry proteomics data have been deposited to the ProteomeXchange Consortium (http://proteomecentral.proteomexchange.org) via the iProX partner repository with the dataset identifier PXD018456 (Ma et al., 2019).

## Ethics Statement

The animal study was reviewed and approved by Animal Ethics Committee of Nanjing University of Chinese Medicine.

## Author Contributions

JY, YL, DZ, and ZZ conceived and designed the experiments and wrote the main text. YL, ZW, LL, and JG contributed to data analysis and manuscript correction. YC, HL, and YY performed herbal preparation and quality control. YT and ZT contributed reagents and materials. J-AD identified the quality of herbs and guided this research. ZW, ZT, and J-AD provided funding support. All authors contributed to the article and approved the submitted version.

## Funding

This work was supported by the National Basic Research Program of China (973 Program, 2011CB505300, 2011CB505303), the National Natural Science Foundation of China (81974525 and 81603357), the 2019 Special Research Plan of Shaanxi Education Department (19JK0229), the School Level Projects of Shaanxi University of Chinese Medicine (2020GP30).

## Conflict of Interest

The authors declare that the research was conducted in the absence of any commercial or financial relationships that could be construed as a potential conflict of interest.
