# Supplementary material for: Licorice-Yuanhua Herbal Pair Induces Ileum Injuries Through Weakening Epithelial and Mucous Barrier Functions: Saponins, Flavonoids, and Di-Terpenes All Involved
Source: Front Pharmacol. 2020 Jul 16;11:869. doi: 10.3389/fphar.2020.00869 (PMC7378851; doi:10.3389/fphar.2020.00869)
Supplement: Supplementary file 1 [file DataSheet_1.pdf]

# Licorice-yuanhua herbal pair induces ileum injuries through weakening epithelial and mucous barrier functions: saponins, flavonoids and di-terpenes all involved

Jingao Yu [1, 2 #], Yanni Liang [1 #], Dongbo Zhang [1 #], Zhen Zhang [1#], Jianming Guo [2], Yanyan Chen [1], Yafeng Yan [1], Hongbo Liu [1], LIyan Lei [1], Zheng Wang [1 *], Zhishu Tang [1 *], Yuping Tang [1], Jin-ao Duan [2 *]

[1] Shaanxi Collaborative Innovation Center of Chinese Medicine Resources Industrialization/ State Key Laboratory of Research & Development of Characteristic Qin Medicine Resources (Cultivation)/ Shaanxi Innovative Drug Research Center/ The Youth Innovation Team of Shaanxi Universities, Shaanxi University of Chinese Medicine, Xianyang, 712000, China; jingao_yu@sina.cn (J.Y.); aiziji_2005@126.com (Y.L.); symensu@163.com (D.Z.); zhzh626@outlook.com (Z.Z.); chenyanyan—c@163.com (Y.C.); 452722757@qq.com (Y.Y.); 15319084280@126.com (H.L.); 2389766835@qq.com (L.L.); yupingtang9@126.com (Y.T.).

[2] Jiangsu Collaborative Innovation Center of Chinese Medicinal Resources Industrialization/ National and Local Collaborative Engineering Center of Chinese Medicinal Resources Industrialization and Formulae Innovative Medicine/ Jiangsu Key Laboratory for High Technology Research of TCM Formulae, Nanjing University of Chinese Medicine, Nanjing, 210023, China; njuguo@njucm.edu.cn (J.G.).

[#] These authors contributed equally to this paper.

* Correspondence: wazh0405@126.com (Zheng Wang); tzs6565@163.com (Zhishu Tang); dja@njucm.edu.cn (Jin-ao Duan).

## Supplementary Informaiton

**Analytical method of UPLC-UV and UPLC-TOF-MS for herb extracts**

Sample preparation: the licorice extracts, yuanhua extracts or the mixture of the two, were dissolved in acetonitrile and centrifuged in 13,000 × g for 10 min, 3 μL supernatant is submitted for analysis.

UPLC conditions: the Waters ACQUITY UPLC system tandem ultraviolet detector (UV) and synapt G2 Q-TOF mass spectrometry were used for chemical profiling analysis. Column: ACQUITY UPLC BEH $C_{18}$ column (100 mm × 2.1 mm, 1.7 μm); Eluents: 0.1 % formic acid solution (A) and acetonitrile (B); Elution procedure: 0- 5 min, 5- 15 % B; 5- 9 min, 15 % B; 9- 30 min, 15- 70 % B; 30- 38 min, 70- 95 % B; 38- 39 min, 95 % B; 39- 40 min, 95- 5 % B. Flow rate: 0.4 mL/min; Column temperature: 35 °C. The wavelength of UV detector was set at 254 nm.

MS conditions: data was acquired in both positive and negative mode, with capillary voltage 3.0 KV, sampling cone voltage 58.0 V, source temperature 120 °C, desolvation temperature 400 °C, desolvation gas flow 900 L/h, and cone gas flow 50 L/h. Scan time was 0.3 second, and mass range 100 to 1200 Da. Leucine-enkephalin solution was used as lock mass material.

Compound identification conditions: after data acquisition, ChromaLynx XS module in the MassLynx 4.1 software was used to identify chemical compounds. Homemade compound databases of licorice and yuanhua were imported into ChromaLynx. Identify method was set as default, excepting the following parameters: retention time 0.5- 40 min; perform spectral deconvolution; retention time tolerance 0.1 min; mass tolerance 5 mDa; peak width auto detection; noise elimination level 6. Reference materials for validation: luteolin (LUT), luteolin-5-*O*-glucoside (LUTG), apigenin (APG), hydroxygenkwanin (HGKW), genkwanin (GKW), tiliroside (TLS), yuanhuapine (YHP), yuanhuacine (YHC), glycyrrhizin (GA) and glycyrrhetinic acid (GRA).

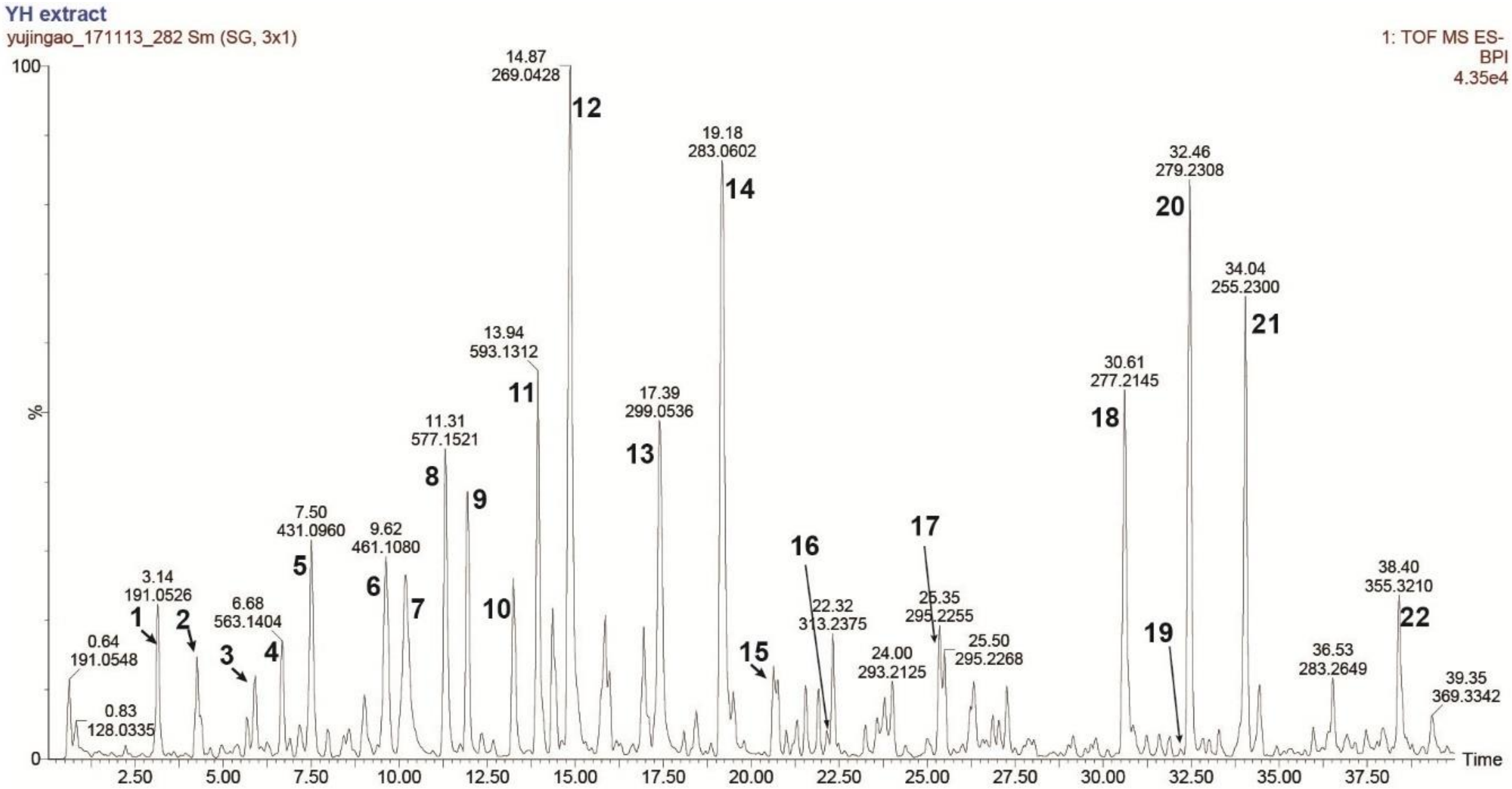


**Figure S1. UPLC-TOF-MS profile of yuanhua extract.** Main chromatographic peaks are identified through matching against a homemade chemical database and/or verified by reference materials. These chemical compounds are: **1**, phenolic acid (possibly Neochlorogenic acid, 3-*O*-Caffeoylquinic acid or 5-*O*-Caffeolyquinic acid); **2**, Cinnamic acid; **3**, Luteolin-5-*O*-glucoside; **4**, Apigenin 6-*C*-glucoside-8-*C*-arabinoside; **5**, flavonoid glycoside (possibly Apigenin 5-β-D-glucopyranoside, Apigenin-

6-*C*-glucoside or Apigenin 7-*O*-β-D-glucopyranoside); **6**, Leucanthoside or 3'-Hydroxygenkwanin 5-*O*-β-D-glucoside; **7**, mix of Magnesium pheophytin and Apigenin-7-*O*-β-D glucuronoside; **8**, flavonoid glycoside (possibly Apigenin 7-rhamnoglucoside, Kaempferitrin, 4',5-dihydroxy-7-methoxy-5-(*O*-xyloglucoside)-flavone, Genkwanin 5-*O*-β-D-primeveroside or Yuenkanin); **9**, Genkwanin 5-*O*-β-D-glucoside; **10**, Luteoline; **11**, Tiliroside; **12**, Apigenin; **13**, Hydroxygenkwanin; **14**, Genkwanin; **15**, fatty acid (possibly 13-Hydroperoxyoctadecadienoic acid, 12-Octadecadienoic acid or 9-Hydroperoxy-10,12-octadecadienoic acid); **16**, Yuanhuapine; **17**, 9-Hydroxy-10,12-octadecadienoic acid; **18**, fatty acid (possibly 5,9,13-Octadecatrienoic acid, Octadecatrienoic acid, Gamma-linolenic acid or 10-Heptadecen-8-ynoic acid methyl ester); **19**, Yuanhuacine; **20**, Octadecadienoic acid; **21**, mainly Myristic acid or 13,13-Dimethyltetradecanoic acid; **22**, Gnidilatin-20-palmitate. Compounds **3, 10, 11, 12, 13, 14, 16, 19** are verified with their reference materials.

**Table S1. Compound identification of Yuanhua extracts through UPLC-TOF-MS technology.** Isomers that cannot be differentiated sufficiently are listed together. Compounds which have been identified with reference materials are marked with red, and chemical types are marked with different background colors.

| No. | Retention time (min) | Positive ion mode | | | Negative ion mode | | | Formula | Candidate compound | | Chemical type |
|---|---|---|---|---|---|---|---|---|---|---|---|
| | | Mass (Da) | Area | Mass Error (mDa) | Mass (Da) | Area | Mass Error (mDa) | | CAS | Name | |
| 1 | 0.63 | - | - | - | 191.0556 | 383.9 | 0.75 | C7H12O6 | 77-95-2 | Quinic acid | phenolic acid |
| 2 | 0.82 | 421.1651 | 8.9 | 0.44 | - | - | - | C25H24O6 | 62596-29-6 | Morusin | flavonoid |
| 3 | 1.48 | - | - | - | 164.0712 | 101.4 | 1.01 | C9H11NO2 | 63-91-2 | Phenylalanine | amino acid |
| 4 | 3.13 | - | - | - | 353.0873 | 318.5 | 2.19 | C16H18O9 | 32719-11-2, 327-97-9, 906-33-2 | Neochlorogenic acid, 3-*O*-Caffeoylquinic acid, 5-*O*-Caffeolyquinic acid | phenolic acid |
| 5 | 3.51 | - | - | - | 367.1545 | 8.8 | 2.13 | C22H24O5 | 1306747-70-5 | Daphnelignan B | lignin |
| 6 | 4.25 | - | - | - | 337.0923 | 242.1 | 1.86 | C16H18O8 | 32451-86-8 | Cinnamic acid | phenolic acid |
| 7 | 4.94 | - | - | - | 367.1029 | 49.1 | 1.86 | C17H20O9 | 123372-74-7, 123410-65-1, 123483-19-2, 40242-06-6 | 4-*O*-Caffeoylquinic acid methyl ester, 5-*O*-Caffeoylquinic acid methyl ester, 3-*O*-Caffeoylquinic acid methyl ester, 5-*O*-Feruloylquinic acid | phenolic acid |
| 8 | 5.21 | 465.1033 | 6.4 | 1.04 | - | - | - | C21H20O12 | 17912-87-7, 482-35-9, 482-36-0 | Myricitrin, Hirsutrin, Hyperin | flavonoid glycoside |
| 9 | 5.45 | 319.0454 | 4.6 | 0.47 | - | - | - | C15H10O8 | 4431-48-5 | Calycopteretin | flavonoid |
| 10 | 5.65 | - | - | - | 347.1158 | 6.6 | 0.98 | C21H18NO4 | 6872-57-7 | Nitidine | alkaloid |
| 11 | 5.93 | 449.1084 | 10.8 | 0.53 | 447.0927 | 437.8 | 1.15 | C21H20O11 | 20344-46-1 | Luteolin-5-*O*-glucoside | flavonoid glycoside |

| | | | | | | | | | | | |
|---|---|---|---|---|---|---|---|---|---|---|---|
| 12 | 6.15 | - | - | - | 579.2078 | 35.5 | 0.67 | C28H36O13 | 137038-13-2, 7374-79-0 | Syringaresinol-4-*O*-β-D-glucopyranoside, Acanthoside B | lignin |
| 13 | 6.16 | - | - | - | 741.2606 | 2.5 | 1.43 | C34H46O18 | 66791-77-3 | Syringaresinol diglucoside | lignin |
| 14 | 6.54 | - | - | - | 417.1186 | 29.9 | 0.74 | C21H22O9 | 1415-73-2 | Barbaloin | anthraquinone |
| 15 | 6.61 | - | - | - | 623.1612 | 17.7 | 1.33 | C28H32O16 | 604-80-8 | Narcissin | flavonoid glycoside |
| 16 | 6.68 | - | - | - | 563.1401 | 421 | 0.24 | C26H28O14 | 51938-32-0 | Apigenin 6-*C*-glucoside-8-*C*-arabinoside | flavonoid glycoside |
| 17 | 6.78 | - | - | - | 521.2023 | 19.2 | 1.51 | C26H34O11 | 107110-16-7 | Lariciresinol 4'-*O*-β-D-glucopyranoside | lignin |
| 18 | 7.51 | - | - | - | 431.0978 | 1756.6 | 1.88 | C21H20O10 | 28757-27-9, 38953-85-4, 578-74-5 | Apigenin 5-β-D-glucopyranoside, Apigenin-6-*C*-glucoside, Apigenin 7-*O*-β-D-glucopyranoside | flavonoid glycoside |
| 19 | 8.70 | 317.0661 | 9.6 | 4.42 | - | - | - | C16H12O7 | 22384-63-0, 571-74-4 | 6-Hydroxyluteolin-7-methyl ether, Herbacetin 8-methyl ether | flavonoid glycoside |
| 20 | 9.60 | - | - | - | 461.1084 | 1803.1 | 0.29 | C22H22O11 | 6980-25-2, 83133-14-6 | Leucanthoside, 3'-Hydroxygenkwanin 5-*O*-β-D-glucoside | flavonoid glycoside |
| 21 | 10.13 | - | - | - | 891.5275 | 4.7 | 2.11 | C55H72MgN4O5 | 479-61-8 | Magnesium pheophytin | other |
| 22 | 10.17 | 447.0927 | 921.4 | 2.4 | - | - | - | C21H18O11 | 29741-09-1 | Apigenin-7-*O*-β-D glucuronoside | flavonoid glycoside |
| 23 | 11.31 | 579.1714 | 187.3 | 0.13 | 577.1557 | 1883.2 | 3.63 | C27H30O14 | 27938-45-0, 482-38-2, 62885-13-6, 77099-20-8, 88423-67-0 | Apigenin 7-rhamnoglucoside, Kaempferitrin, 4',5-dihydroxy-7-methoxy-, 5-(*O*-xyloglucoside)-flavone, Genkwanin 5-*O*-β-D-primeveroside, Yuenkanin | flavonoid glycoside |
| 24 | 11.69 | - | - | - | 607.1663 | 49.3 | 0.4 | C28H32O15 | 520-27-4 | Diosmin | flavonoid glycoside |
| 25 | 11.74 | - | - | - | 541.1135 | 117.1 | 1.29 | C30H22O10 | 105227-18-7, 111103-90-3, 159736-35-3, 160963-92-8, 163565-71-7, 163660-10-4, 178897-28-4, 215868-02-3, 871319-96-9, 90411-12-4, 90411-13-5, 93413-00-4, 93859-63-3, 95733-02-1, 958640-20-5, 958640-21-6, 1326705-92-3, 142674-67-7, 151283-11-3, 178664-65-8, 178897-27-3 | Chamaejasmine, Genkwanol A, Wikstrol A, Wikstrol B, Daphnodorin E, Daphnodorin F, Daphnodorin I, Daphnodorin M, Isoneochamaejasmin A, Neochamaejasmin B, Neochamaejasmin A, Chamaechromone, Isochamaejasmin, Daphnodorin B, Daphnogirin A, Daphnogirin B, 2'''-Dehydroxy-3,3''-bisteppogenin, Genkwanol B, Genkwanol C, Daphnodorin G, Daphnodorin H | diterpine |
| 26 | 11.93 | - | - | - | 445.1135 | 1877.2 | 0.04 | C22H22O10 | 552-52-3 | Genkwanin 5-*O*-β-D-glucoside | flavonoid glycoside |
| 27 | 12.67 | - | - | - | 543.1291 | 143.3 | 0.89 | C30H24O10 | 1402045-66-2, 190078-00-3 | 3''-epi-Dihydrodaphnodorin B, Dihydrodaphnodorin B | phenylpropanoid |

| | | | | | | | | | | | |
|---|---|---|---|---|---|---|---|---|---|---|---|
| 28 | 13.25 | 287.0556 | 647.4 | 1.52 | 285.0399 | 1562.6 | 1.46 | C15H10O6 | 491-70-3 | Luteoline | flavonoid |
| 29 | 13.70 | - | - | - | 549.1972 | 18.7 | 0.86 | C27H34O12 | 99633-12-2 | Eucommin A | lignin |
| 30 | 13.94 | 595.1452 | 43.5 | 2.1 | 593.1295 | 2911.6 | 1.71 | C30H26O13 | 20316-62-5 | Tiliroside | flavonoid glycoside |
| 31 | 13.99 | - | - | - | 657.1456 | 21.6 | 1.11 | C31H30O16 | 119179-04-3 | Rutarensin | coumarin |
| 32 | 14.86 | - | - | - | 269.0450 | 6449.5 | 2.15 | C15H10O5 | 520-36-5 | Apigenin | flavonoid |
| 33 | 14.88 | - | - | - | 557.1084 | 229.7 | 1.15 | C30H22O11 | 1326705-92-3, 142674-67-7, 151283-11-3, 178664-65-8, 178897-27-3 | 2'''-Dehydroxy-3,3''-bisteppogenin, Genkwanol B, Genkwanol C, Daphnodorin G, Daphnodorin H | flavonoid |
| 34 | 15.23 | - | - | - | 355.1182 | 30.2 | 0.43 | C20H20O6 | 133644-85-6, 156974-99-1, 28071-34-3, 28115-68-6, 54983-95-8, 83708-70-7, 85404-48-4 | Dihydrosesamin, Isosalicifoline, Dihydrosesamin, Pluviatolide, Xanthoxylol, Dihydrosesamin, Isopluviatolide | phenylpropanoid |
| 35 | 17.40 | 301.0712 | 346.3 | 2.4 | 299.0556 | 1983.7 | 1.97 | C16H12O6 | 20243-59-8 | Hydroxygenkwanin | flavonoid |
| 36 | 18.68 | - | - | - | 401.1865 | 8.2 | 0.77 | C25H26N2O3 | 58115-31-4 | Aurantiamide | amide |
| 37 | 19.19 | 285.0763 | 369.8 | 1.56 | 283.0606 | 6665.3 | 0.41 | C16H12O5 | 437-64-9 | Genkwanin | flavonoid |
| 38 | 19.49 | - | - | - | 313.0712 | 496.8 | 1.3 | C17H14O6 | 25739-41-7, 32174-62-2, 3301-49-3 | Luteolin 3',7-dimethyl ether, Luteolin 7,4'-dimethyl ether, Kaempferol 3,7-*O*-dimethyl ether | flavonoid |
| 39 | 20.07 | - | - | - | 351.0869 | 7.2 | 2.09 | C20H16O6 | 493-95-8 | Hibalactone | lignin |
| 40 | 20.62 | - | - | - | 559.2179 | 112.6 | 0.26 | C29H36O11 | 845882-85-1 | Genkwanine L | diterpine |
| 41 | 20.67 | - | - | - | 311.2222 | 1148.5 | 0.42 | C18H32O4 | 23017-93-8, 63121-49-3, 7324-20-1 | 13-Hydroperoxyoctadecadienoic acid, 12-Octadecadienoic acid, 9-Hydroperoxy-10,12-octadecadienoic acid | fatty acid |
| 42 | 20.99 | - | - | - | 607.2543 | 190.2 | 0.5 | C34H40O10 | 845882-77-1, 845882-81-7 | Genkwanine D, Genkwanine H | fatty acid |
| 43 | 21.88 | - | - | - | 485.2175 | 91.8 | 1.3 | C27H34O8 | 147253-46-1 | Daphnetoxin | diterpine |
| 44 | 22.15 | 543.2230 | 74.4 | 1.34 | 541.2074 | 169.8 | 0.45 | C29H34O10 | 104901-03-3 | Yuanhuapine | diterpine |
| 45 | 22.36 | - | - | - | 491.2658 | 28.9 | 1.99 | C28H36N4O4 | 38840-26-5 | Mucronine B | alkaloid |
| 46 | 23.08 | 299.0919 | 5.3 | 1.95 | - | - | - | C17H14O5 | 34086-51-6, 5128-44-9 | Apigenin 7,4'-dimethyl ether, 5-Hydroxy-4',7-dimethoxyisoflavone | flavonoid |
| 47 | 25.36 | - | - | - | 295.2273 | 963.6 | 1.83 | C18H32O3 | 15514-85-9 | 9-Hydroxy-10,12-octadecadienoic acid | fatty acid |
| 48 | 26.40 | - | - | - | 653.3326 | 22.6 | 0.42 | C37H50O10 | 1493767-50-2, 1499166-94-7, 1895064-33-1, 1895064-34-2, 845882-75-9, 49845882-79-3 | Genkwanine T, Genkwanine U, Neogenkwanine C, Neogenkwanine D, Genkwanine B, Genkwanine F | diterpine |
| 49 | 26.79 | - | - | - | 665.2962 | 7.6 | 0.57 | C37H46O11 | 1228542-56-0 | Yuanhuaoate E, | diterpine |

| | | | | | | | | | | | |
|---|---|---|---|---|---|---|---|---|---|---|---|
| 50 | 27.72 | - | - | - | 555.2958 | 25 | 2.03 | C32H44O8 | 1366053-73-7, 16561-27-6 | Prostratin Q, 12-*O*-Decanoylphorbol 13-acetate | diterpine |
| 51 | 28.76 | 695.3431 | 37.4 | 2.44 | 693.3275 | 6.7 | 0.46 | C39H50O11 | 1127258-68-7 | Hirsein B | diterpine |
| 52 | 30.61 | - | - | - | 277.2168 | 2726.5 | 2.31 | C18H30O2 | 119984-95-1, 27213-43-0, 506-26-3, 871843-96-8 | 5,9,13-Octadecatrienoic acid, Octadecatrienoic acid, Gamma-linolenic acid, 10-Heptadecen-8-ynoic acid, methyl ester | fatty acid |
| 53 | 30.62 | - | - | - | 277.0865 | 2.4 | 0.53 | C18H14O3 | 67656-29-5 | Methylenetanshinquinone | other |
| 54 | 30.65 | - | - | - | 455.3525 | 100.9 | 1.9 | C30H48O3 | 77-52-1 | Ursolic acid | triterpene |
| 55 | 32.20 | 649.3013 | 110.5 | 0.06 | 647.2856 | 26.4 | 2.91 | C37H44O10 | 60195-70-2 | Yuanhuacin | diterpine |
| 56 | 32.46 | - | - | - | 279.2324 | 4327.3 | 1.57 | C18H32O2 | 28984-77-2 | Octadecadienoic acid | fatty acid |
| 57 | 33.29 | - | - | - | 305.2481 | 201.7 | 1.63 | C20H34O2 | 1191-41-9 | Ethyl linolenate | fatty acid |
| 58 | 34.04 | - | - | - | 255.2324 | 2660 | 2.44 | C16H32O2 | 124-06-1, 32013-53-9 | Myristic acid, 13,13-Dimethyltetradecanoic acid | fatty acid |
| 59 | 34.04 | - | - | - | 357.1702 | 16 | 0.88 | C21H26O5 | 68143-83-9 | Virolin | diterpine |
| 60 | 35.36 | - | - | - | 269.2481 | 56.7 | 2.42 | C17H34O2 | 506-12-7 | Margaric acid | fatty acid |
| 61 | 36.63 | - | - | - | 619.2920 | 26.5 | 0.01 | C37H40N4O5 | 55100-98-6 | Pheophorbide a, ethyl ester | other |
| 62 | 37.26 | 635.3220 | 1379 | 0.23 | - | - | - | C37H46O9 | 1360473-89-7 | Acutilobin F | coumarin ester |
| 63 | 38.43 | 891.5622 | 2.7 | 2.15 | - | - | - | C53H78O11 | 60195-67-7 | Gnidilatin-20-palmitate | Diterpine ester |
| 64 | 38.63 | - | - | - | 431.3161 | 126 | 1.32 | C27H44O4 | 69573-60-0, 872139-53-2, 952062-20-3 | Caffeic acid *n*-octadecyl ester, Octadecyl caffeate, *Z*-Octadecyl caffeate | fatty acid ester |

“-” not detected/ mass error out of range.

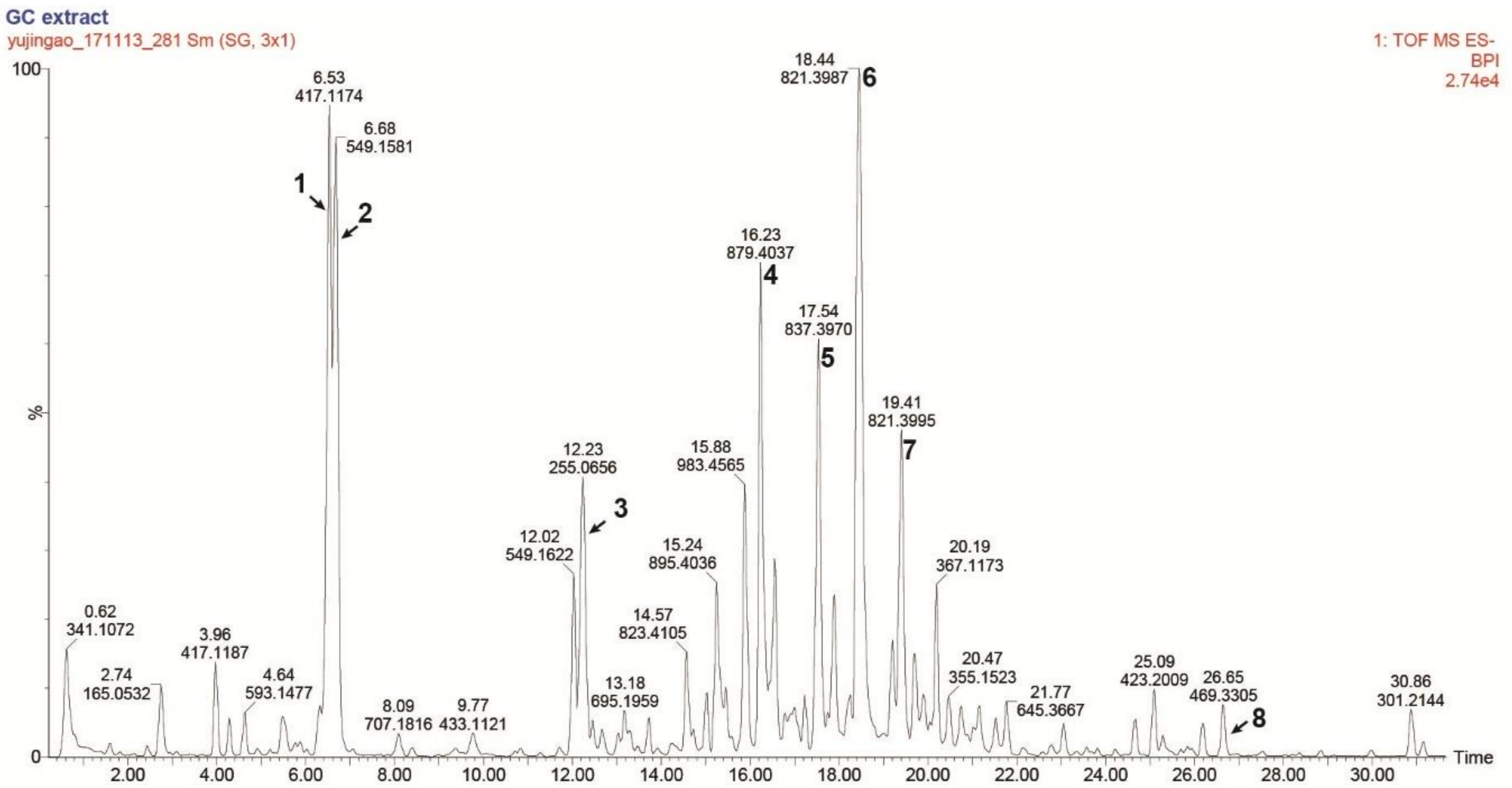


**Figure S2. UPLC-TOF-MS profile of licorice extract.** Main chromatographic peaks are identified as: **1**, Liquiritin or Isoliquiritin; **2**, Liquiritin apioside or Isoliquiritin apioside; **3**, flavonoid (possibly Liquiritigenin, Isoliquiritigenin, or Pinocembrin); **4**, 22-Acetoxyl-glycyrrhizin; **5**, triterpenoid saponin (possibly Yunganoside K2, Licorice saponin G2 or 22-Hydroxy-glycyrrhizin); **6**, Glycyrrhizin; **7**, triterpenoid saponin (possibly Licorice saponin H2,Licorice saponin K2, 18α-Glycyrrhizin,

Uralsaponin B, Yunganoside L2, Yunganoside J2); **8**, Glycyrrhetinic acid. Compounds **6** and **8** are verified with their reference materials.

**Table S2. Compound identification of licorice extract through UPLC-TOF-MS technology.** Isomers that cannot be differentiated sufficiently are listed together. Compounds identified with reference materials are marked with red, and chemical types are marked with different background colors.

| No. | Retention time (min) | Positive ion mode | | | Negative ion mode | | | Formula | Candidate compound | | Chemical type |
|---|---|---|---|---|---|---|---|---|---|---|---|
| | | Mass (Da) | Area | Mass Error (mDa) | Mass (Da) | Area | Mass Error (mDa) | | CAS | Name | |
| 1 | 3.97 | - | - | - | 579.1714 | 9.6 | 0.25 | C27H32O14 | 10236-47-2 | Naringin | flavonoid glycoside |
| 2 | 4.64 | 595.1663 | 11.6 | 1.26 | 593.1506 | 107.6 | 0.74 | C27H30O15 | 482-39-3, 17650-84-9, 23666-13-9, 40581-17-7 | Kaempferin, Nicotiflorin, Vicenin-2, Violanthin | flavonoid glycoside |
| 3 | 5.48 | - | - | - | 563.1401 | 162.7 | 2.08 | C26H28O14 | 52012-29-0, 51938-32-0 | Isoschaftoside, Schaftoside | flavonoid glycoside |
| 4 | 5.88 | 549.1608 | 6.8 | 0.81 | - | - | - | C26H28O13 | 199796-12-8 | Liquiritigenin-7-*O*-β-D-apiofuranosyl-4'-*O*-β-D-glucopyranoside | flavonoid glycoside |
| 5 | 6.53 | - | - | - | 417.1186 | 3064 | 0.06 | C21H22O9 | 5041-81-6, 551-15-5 | Isoliquiritin, Liquiritin | flavonoid glycoside |
| 6 | 6.68 | - | - | - | 549.1608 | 1771 | 2.92 | C26H30O13 | 120926-46-7, 74639-14-8 | Isoliquiritin apioside, Liquiritin apioside | flavonoid glycoside |
| 7 | 6.77 | 579.1714 | 14.3 | 1.23 | - | - | - | C27H30O14 | 40788-84-9 | Isoviolanthin | flavonoid |
| 8 | 9.62 | 301.0712 | 8.2 | 1.05 | - | - | - | C16H12O6 | 491-71-4, 1592-70-7 | Chrysoeriol, Kaempferol-3-*O*-methyl ether | flavonoid |
| 9 | 10.53 | 271.0606 | 26.6 | 2.24 | - | - | - | C15H10O5 | 20575-57-9, 548-83-4, 446-72-0, 1592-70-7 | Calycosin, Galangin, Genistein, Isokaempferide | flavonoid |
| 10 | 12.23 | - | - | - | 255.0657 | 1358 | 1.42 | C15H12O4 | 961-29-5, 578-86-9, 480-39-7 | Isoliquiritigenin, Liquiritigenin, Pinocembrin | flavonoid |
| 11 | 12.99 | - | - | - | 985.4644 | 8.4 | 0.59 | C48H74O21 | 142545-75-3 | Yunganoside D1, Yunganoside G1 | flavonoid glycoside |
| 12 | 13.18 | - | - | - | 695.1976 | 133.4 | 1.73 | C35H36O15 | not available | Licorice glycoside D1, Licorice glycoside D2 | flavonoid glycoside |
| 13 | 13.3 | 287.0556 | 10.4 | 2.34 | - | - | - | C15H10O6 | 520-18-3, 480-23-9 | Kaempferol, Orobol | flavonoid |
| 14 | 13.46 | - | - | - | 839.4065 | 38.5 | 1.86 | C42H64O17 | not available | Yunganoside G2 | flavonoid glycoside |
| 15 | 13.94 | - | - | - | 563.1765 | 5.2 | 1.11 | C27H32O13 | 5041-81-6 | Isoliquiritoside | flavonoid glycoside |
| 16 | 14.34 | - | - | - | 692.1979 | 31.1 | 0.91 | C35H35O14N | not available | Licorice glycoside E | flavonoid glycoside |
| 17 | 14.58 | 455.3525 | 490.9 | 0.29 | - | - | - | C30H46O3 | 17991-81-0 | Glypallidifloric acid | triterpenoid |
| 18 | 14.58 | - | - | - | 823.4116 | 330 | 1.15 | C42H64O16 | not available | Licorice saponin J2, Uralsaponin C | flavonoid glycoside |

| | | | | | | | | | | | |
|---|---|---|---|---|---|---|---|---|---|---|---|
| 19 | 15.01 | - | - | - | 999.4437 | 121.5 | 1.87 | C48H72O22 | 1262326-47-5 | 24-Hydroxy-licorice-saponin A3 | triterpenoid saponin |
| 20 | 15.46 | - | - | - | 853.3858 | 157.3 | 4.96 | C42H62O18 | 118441-84-2 | 22-Hydroxy-licorice-saponin G2 | triterpenoid saponin |
| 21 | 15.9 | - | - | - | 983.4488 | 277.3 | 2.51 | C48H72O21 | 118325-22-7, 156980-50-6 | Licorice saponin A3, Yunganoside K1 | triterpenoid saponin |
| 22 | 16.23 | - | - | - | 879.4014 | 1101 | 2.25 | C44H64O18 | not available | 22-Acetoxyl-glycyrrhizin | triterpenoid |
| 23 | 16.26 | 511.3424 | 3452 | 0.52 | - | - | - | C32H46O5 | 6277-14-1 | 3β-Acetylglycyrrhetic acid | triterpenoid |
| 24 | 16.47 | 837.3909 | 22 | 0.56 | - | - | - | C42H60O17 | 1262326-48-6, 1262489-45-1 | 24-Hydroxyl-licorice, Uralsaponin D | triterpenoid saponin |
| 25 | 16.56 | 469.3318 | 454.2 | 0.24 | - | - | - | C30H44O4 | 10301-75-4, 10401-33-9, 131559-55-2 | 3-oxo-18β-Glycyrrhetinic acid, glabrolide, glyyunnansapogenin C | triterpenoid |
| 26 | 17.18 | - | - | - | 951.4590 | 6.3 | 0.28 | C48H72O19 | not available | Licorice-saponin F3 | triterpenoid saponin |
| 27 | 17.52 | 839.4065 | 163 | 2.9 | - | - | - | C42H62O17 | 156980-51-7, 118441-84-2 | Yunganoside K2, Licorice saponin G2, 22-Hydroxy-glycyrrhizin | triterpenoid saponin |
| 28 | 18.45 | 823.4116 | 377.8 | 2.31 | 821.3960 | 1883 | 1.0 | C42H62O16 | 1405-86-3 | Glycyrrhizin | triterpenoid saponin |
| 29 | 19.41 | - | - | - | 821.3995 | 835.2 | 2.5 | C42H62O16 | 118441-85-3, 83896-44-0, 105038-43-5 | Licorice saponin H2,Licorice saponin K2, 18α-Glycyrrhizin, Uralsaponin B, Yunganoside L2, Yunganoside J2 | triterpenoid saponin |
| 30 | 19.9 | - | - | - | 353.1025 | 34.2 | 0.25 | C20H18O6 | 117038-80-9 | Licopyranocomarin | coumarin |
| 31 | 20.19 | - | - | - | 367.1182 | 707.3 | 0.85 | C21H20O6 | 122290-50-0, 124596-86-7, 129145-52-4, 161099-37-2, 125709-32-2, 156162-05-9, 94805-82-0, 197304-07-7, 117038-82-1 | 7-*O*-Methylluteone, Gancaonin B, Gancaonin N, Glicoricone, Glisoflavone, Glyasperin M, Glycycoumarin, Glycyrrhiza-30isoflavones B, Isoglycycoumarin | flavonoid or coumarin |
| 32 | 20.5 | - | - | - | 805.4010 | 175.8 | 0.78 | C42H62O15 | 118525-49-8 | Licorice saponin C2 | triterpenoid saponin |
| 33 | 21.05 | 353.1389 | 40.3 | 2.00 | - | - | - | C21H20O5 | not available | Glabroisoflavanone B | flavonoid |
| 34 | 21.53 | - | - | - | 381.1338 | 161.2 | 1.49 | C22H22O6 | 23013-85-6, 23013-84-5, 156250-69-0, 51847-92-8 | 3-*O*-Methyl-glycyrol, 5-*O*-Methyl-glycyrol, Kanzonol O, Licoricone | flavonoid |
| 35 | 22.6 | - | - | - | 323.1283 | 17.7 | 0.08 | C20H20O4 | 886620-61-7 | Abyssinone II | flavonoid |
| 36 | 24.21 | - | - | - | 391.1909 | 19.9 | 0.75 | C25H28O4 | 59870-65-4, 68978-02-9 | Glabrol, Hispaglabridin B | coumarin |
| 37 | 24.67 | - | - | - | 369.1702 | 143.3 | 1.74 | C22H26O5 | 142561-10-2 | Glyasperins D | coumarin |
| 38 | 25.39 | - | - | - | 407.1858 | 11.2 | 1.98 | C25H28O5 | 142542-83-4 | Glyinflanin A | flavonoid |
| 39 | 25.69 | - | - | - | 421.1651 | 30 | 0.41 | C25H26O6 | 66777-70-6, 199331-53-8, 142474-52-0, 199331-53-8 | 6,8-Diprenyl orobol, Glyarallins B, Glyasperin A, Glyuralins B | flavonoid |
| 40 | 25.94 | - | - | - | 423.1808 | 23.1 | 0.02 | C25H28O6 | 124596-89-0, 129280-34-8 | 3'-(γ,γ-dimethylallyl)-kievitone, Gancaonin E, | flavonoid |

| | | | | | | | | | | | |
|---|---|---|---|---|---|---|---|---|---|---|---|
| | | | | | | | | | | Glisoflavanone, Glyinflanin E, Glyinflanin F, Isoangustone A | |
| 41 | 26.26 | - | - | - | 437.1600 | 9.7 | 1.46 | C25H26O7 | 181476-22-2 | Kanzonol T | flavonoid |
| 42 | 26.64 | - | - | - | 469.3318 | 239.7 | 1.31 | C30H46O4 | 471-53-4 | Glycyrrhetinic acid | triterpenoid |
| 43 | 27.55 | - | - | - | 405.1702 | 10.6 | 1.04 | C25H26O5 | 134958-52-4, 142750-24-1, 220860-37-7 | Gancaonin Q, Glyinflanin C, Kanzonol Z | flavonoid |
| 44 | 29.99 | - | - | - | 455.3525 | 28.3 | 0.11 | C30H48O3 | 472-15-1, 14226-18-7 | Betulinic acid, Glycyrrhetol | triterpenoid |

“-” not detected/ mass error out of range.

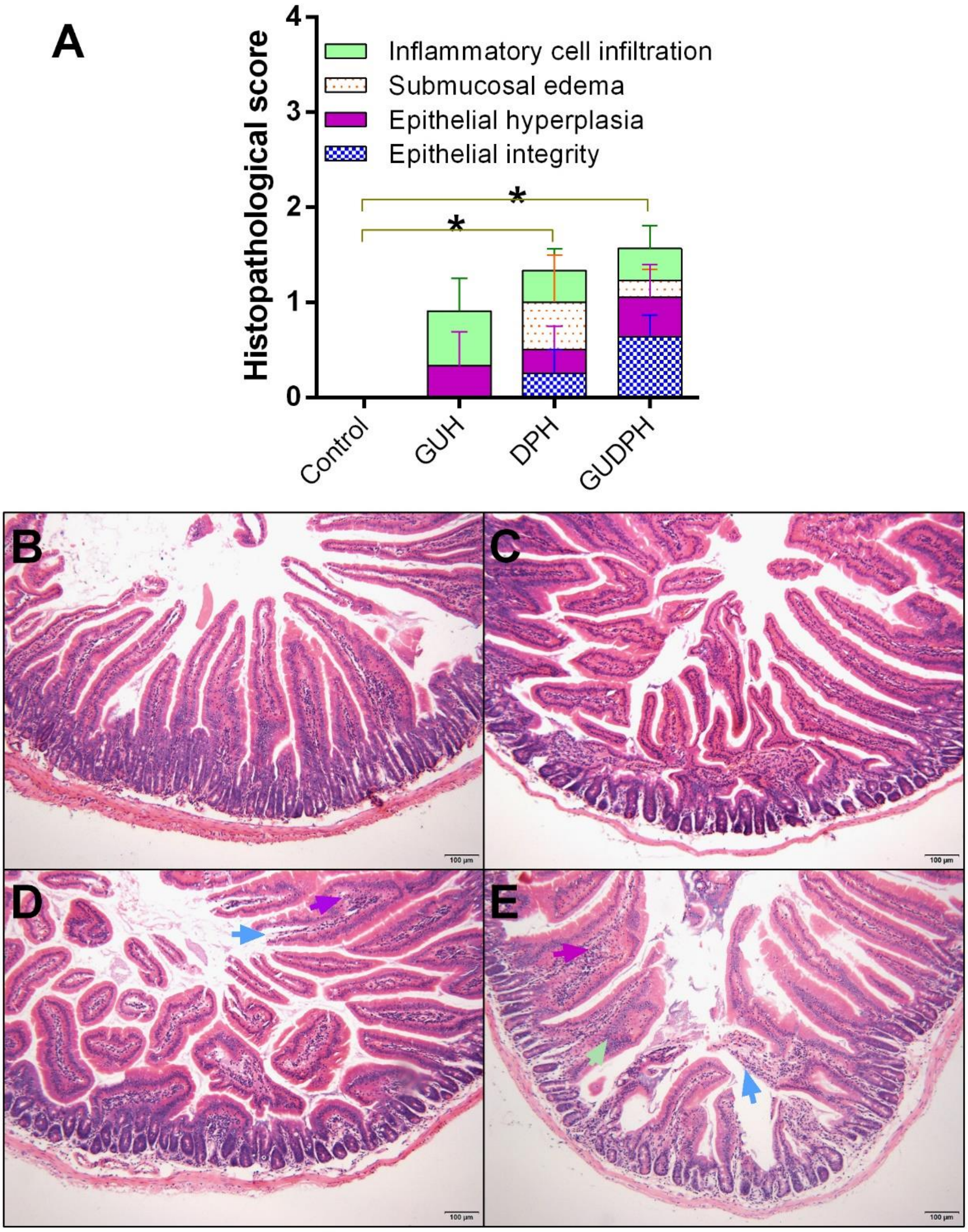


**Figure S3. Histopathological staining and evaluation of mice duodenum.** (A) Bar chart of histopathological scores of ileum tissues in each group. * P< 0.05 compared with control group, indicated by connecting lines. (B-E) HE staining of mice duodenum tissues in Control, GUH, DPH and GUDPH groups, respectively. Blue arrows are signs of epithelial integrity loss, purple arrows are signs of submucosal edema and hyperplasia, and green arrows are signs of inflammation cell infiltration. GUH, licorice high dose; DPH, yuanhua high dose; GUDPH, herbal pair high dose.

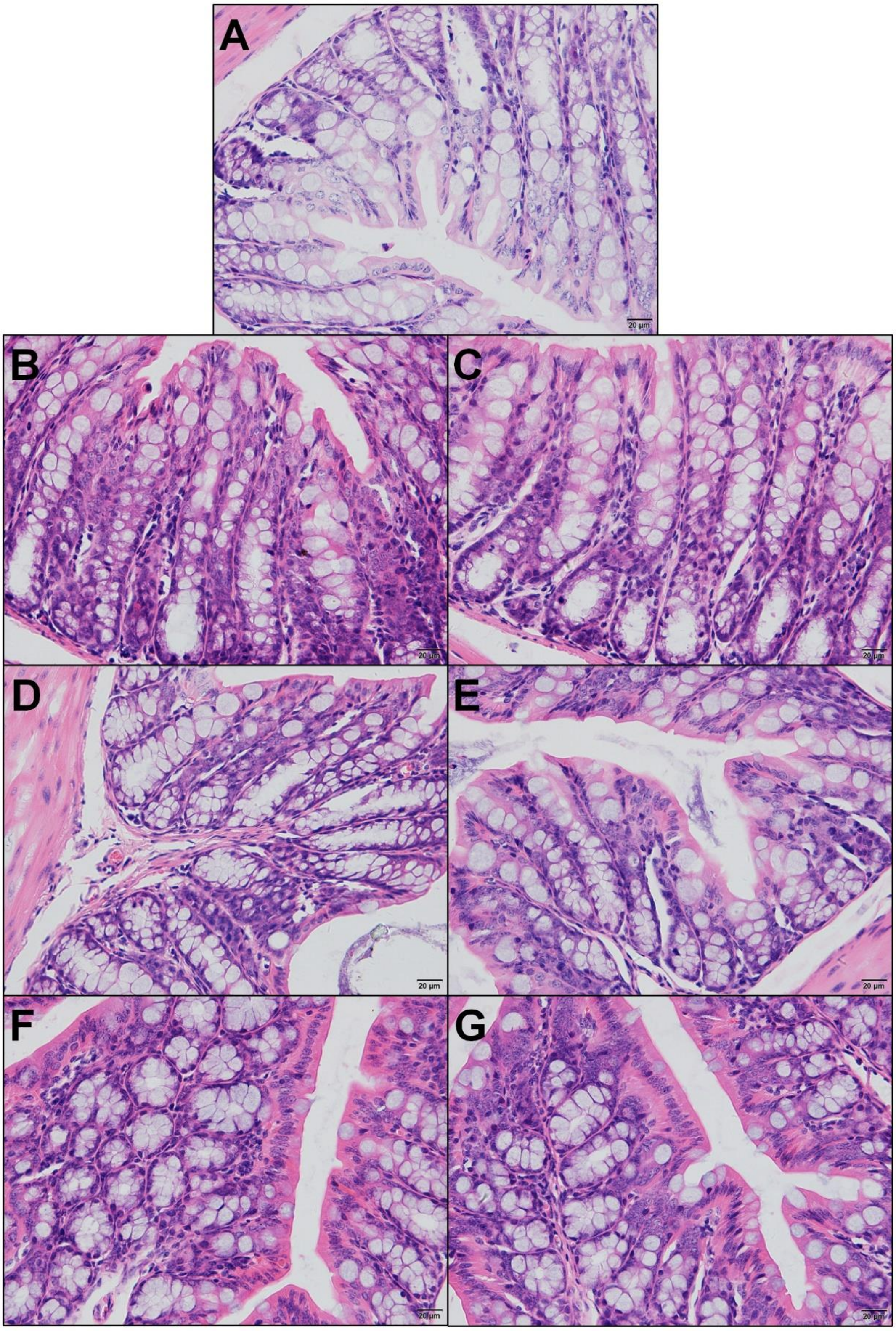


**Figure S4. Histopathological staining of mice colon.** (A-G) HE staining of mice colon tissues in Control, GUH, GUL, DPH, DPL, GUDPH and GUDPL groups, respectively. No signs of damages are found. White holes in each image represent mucus in goblet cells. GUH, licorice high dose; GUL, licorice low dose; DPH, yuanhua high dose; DPL, yuanhua low dose; GUDPH, herbal pair

high dose; and GUDPL, herbal pair low dose.

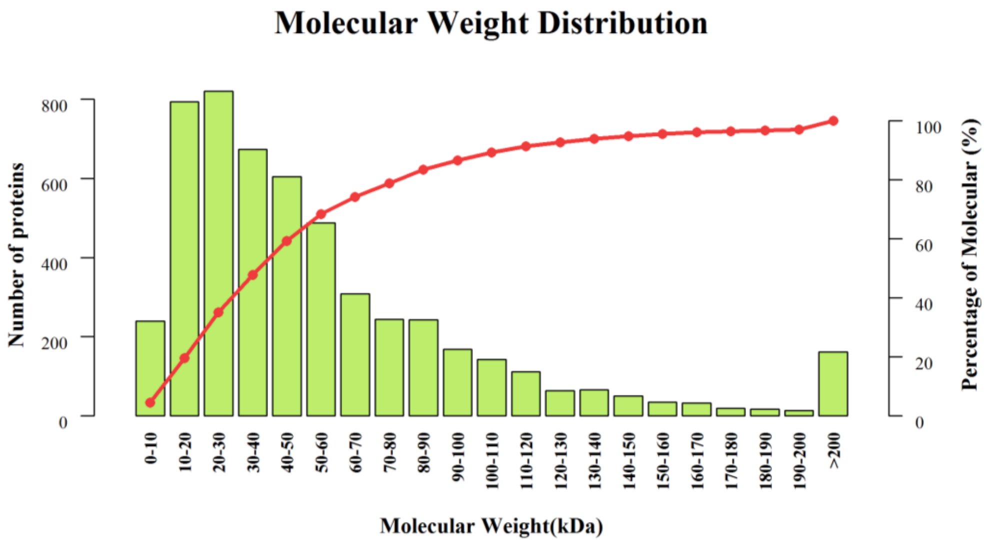


**Figure S5. Frequency distribution of molecular weight of proteins detected by iTRAQ-labeled proteomic experiments.**

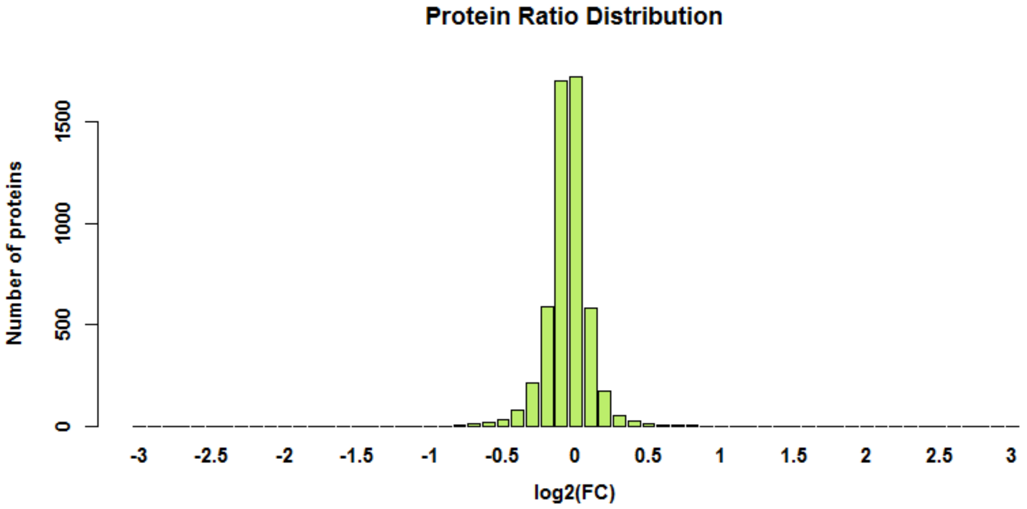


**Figure S6. Fold change of proteins detected by iTRAQ-labeled proteomic experiments. Fold changes are converted by the "log2()" function.**

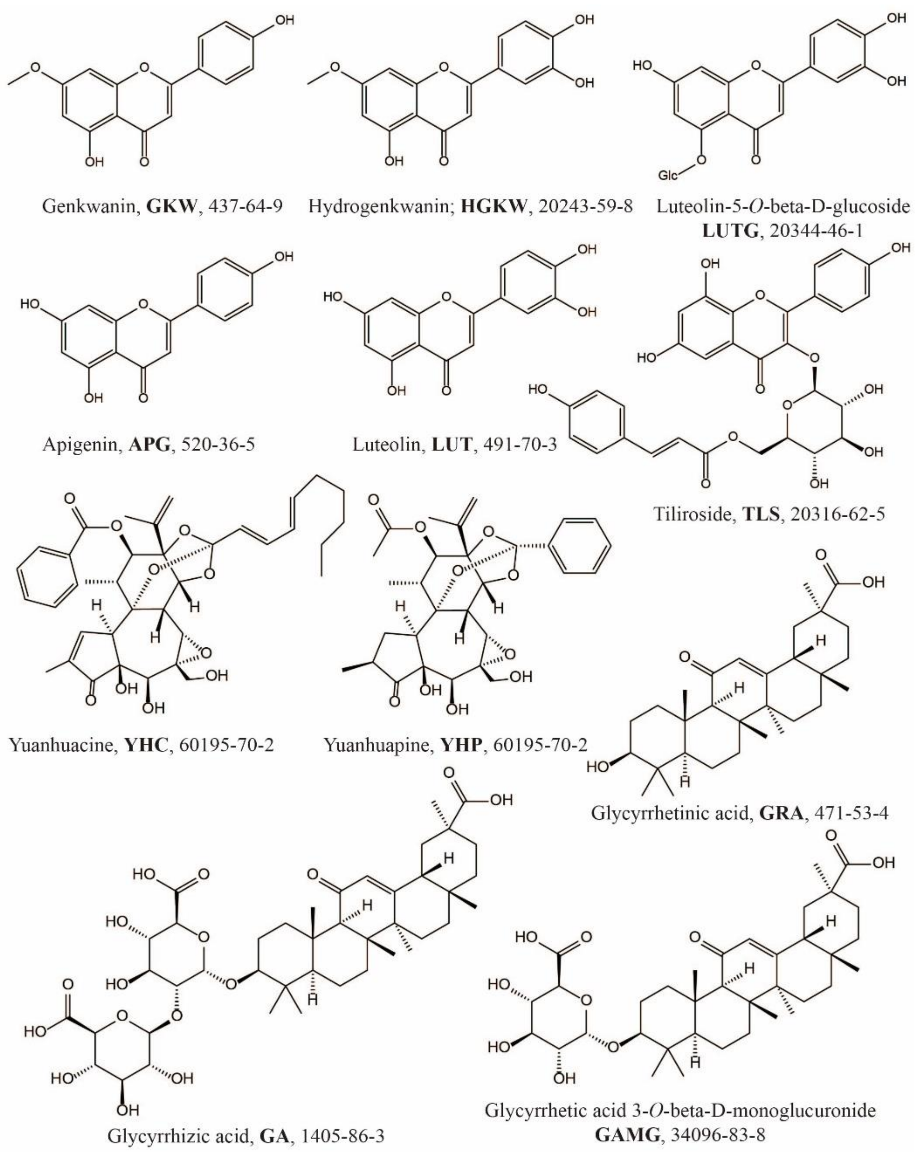


**Figure S7. Chemical structures of representative saponins and flavonoids in licorice-yuanhua herbal pair.** Under each structure, chemical names, abbreviations and CAS numbers are given.
